# Gaussian network model can be enhanced by combining solvent accessibility in proteins

**DOI:** 10.1038/s41598-017-07677-9

**Published:** 2017-08-08

**Authors:** Hua Zhang, Tao Jiang, Guogen Shan, Shiqi Xu, Yujie Song

**Affiliations:** 1 School of Computer and Information Engineering, Zhejiang Gongshang University, Hangzhou Zhejiang, P.R. China 310018; 2 School of Statistics and Mathematics, Zhejiang Gongshang University, Hangzhou Zhejiang, P.R. China 310018; 3 School of Community Health Sciences, University of Nevada Las Vegas, Las Vegas, NV 89154 USA

## Abstract

Gaussian network model (GNM), regarded as the simplest and most representative coarse-grained model, has been widely adopted to analyze and reveal protein dynamics and functions. Designing a variation of the classical GNM, by defining a new Kirchhoff matrix, is the way to improve the residue flexibility modeling. We combined information arising from local relative solvent accessibility (RSA) between two residues into the Kirchhoff matrix of the parameter-free GNM. The undetermined parameters in the new Kirchhoff matrix were estimated by using particle swarm optimization. The usage of RSA was motivated by the fact that our previous work using RSA based linear regression model resulted out higher prediction quality of the residue flexibility when compared with the classical GNM and the parameter free GNM. Computational experiments, conducted based on one training dataset, two independent datasets and one additional small set derived by molecular dynamics simulations, demonstrated that the average correlation coefficients of the proposed RSA based parameter-free GNM, called RpfGNM, were significantly increased when compared with the parameter-free GNM. Our empirical results indicated that a variation of the classical GNMs by combining other protein structural properties is an attractive way to improve the quality of flexibility modeling.

## Introduction

Proteins are not static but are constantly in motion^1^. The structural flexibility and dynamics associated with these motions allows conformational changes to implement various important biological processes and functions^2–5^. Experimentally available X-ray structures provide information on the atomic mobility, also known as the Debye–Waller temperature factor or B-factor. This parameter is proportional to the mean square displacement in a crystal due to atomic mobility and positional disorder. As a dynamics parameter, the B-factor has been widely examined, including the relationship between mobility and thermal stability^6, 7^, understanding various protein function^4, 5, 8, 9^, and in the context of the evaluation on flexibility modeling^10, 11^, etc. Consequently, accurate predictions of B-factors offer a good starting point for understanding the relationship between protein structures and functions.

Several physical and computational models have been proposed to predict the B-factors from electron density maps^12^, protein structures^10, 11^, and sequences^13–16^. Besides, molecular dynamic (MD) simulation may have the ability to in detail investigate the dynamic link between protein structures and functions. However, the drawback of MD simulations is the their high computational cost^17^. Therefore, many structure-based computational approaches were developed, such as normal mode analysis^18–20^, elastic network model (ENM)^21^, packing density^11^, and weighted contact number^22^. The ENMs, including the isotropic GNM (Gaussian network model)^23–25^ and the ANM (anisotropic network model)^26^, define spring-like interactions between residues that are within a certain cutoff distance. They simplify the complicated all-atom potentials into a quadratic function in the vicinity of the equilibrium state, which allows for decomposing the motions into normal modes with different frequencies. They can determine the (concerted) collective motions of residues that correspond to the lowest-frequency modes comprising large parts of a given protein^27^. Due to the simplicity and the efficiency, ENMs (GNM and ANM) have been validated in numerous applications that were resulted in reasonable agreement with a wealth of experimental data, including prediction of X-ray crystallographic B-factors for amino acids^16, 24^, identifications of functional sites^28, 29^, elucidation of the molecular mechanisms of motor-protein motions^30^, and general conformational changes and functions^3, 5, 31–43^.

The classical GNM^24, 26^, regarded as the simplest coarse-grained model, defines spring-like interactions between C_α_ or C_β_ atoms of residues within a certain cutoff distance. An arbitrary cutoff distance delimits the range of interactions and different cutoffs may generate non-unique outcomes. Moreover, several variations of GNMs have been developed to improve the modeling of protein dynamics^10, 44–46^. Particularly, Yang *et al*.^45^ developed a parameter-free Gaussian network model (pfGNM) that replaced the distance cutoff using the inverse square distance. It significantly improved the B-factor prediction when compared with the classical GNM^45^. Alternatively, the structure-based method DsspRSA9 in our previous work^14^, which investigated the relationship between the residue flexibility measured using B-factor and the local solvent accessibility, can provide better prediction of B-factors when compared with pfGNM^14, 16^. The relative solvent accessibility (RSA) measures the solvent exposure that is defined as the accessible surface area (ASA) of a residue accessible to a solvent normalized by the ASA of this residue in its extended tripeptide (Ala-X-Ala) conformation^47^. The RSA based method DsspRSA9 is a relatively simple model that utilized linear regression to fit the B-factors using actual RSA values in the sliding window of the central residue with a size of 9. However, the drawback is the fact that DsspRSA9 cannot provide normal modes as well as the information about the collective motions in contrast to GNM and pfGNM.

Inspired by the gap in B-factor prediction quality between pfGNM and DsspRSA9, we proposed a variation of the parameter-free Gaussian network model, called RpfGNM, by adding the information from the relative solvent accessibility of residues. The proposed model aims to combine the advantages of the pfGNM and the RSA-based method to improve the B-factor predictions. Meanwhile, the proposed RpfGNM provides normal modes as well as information about the collective motions.

## Materials and Methods

### Benchmark datasets

We used a benchmark dataset which was previously used in Zhang *et al*.^14^ and filtered using PDB-REPRDB^48^. This set was composed of 972 protein chains extracted from the Protein Data Bank (PDB)^49^ with length ≥ 60, sequence identity ≤ 25%, and high-quality X-ray structures to derive reliable native B-factors (resolution ≤ 2.0 Å and R-factor ≤ 0.2). The lengths of protein sequences in PDB972 range from 60 to 1491. Performing GNM for one protein will become time-consuming due to the inverse matrix computation in GNM as the sequence length increases. To fasten the whole learning procedure, we estimated the undetermined parameters associated with the solvent accessibility information based on a subset of the dataset PDB972. This subset, called PDB365, includes 365 chains that are composed of protein sequences with a length ≤ 200. The remaining set, denoted by PDB607, includes 607 chains with a sequence length > 200. Note that the dataset PDB972 is actually the union of PDB365 and PDB607. Similarly as in the studies by Zhang *et al*.^14^ and Yang *et al*.^45^, the average correlation coefficient (ACC) was adopted to assess the performance of models.

We also prepared an independent dataset with low sequence identity with PDB927. This dataset comprised sequences solved by X-ray crystallography and deposited in PDB between Jan. 2010 and Sept. 2015, which was after PDB972 dataset being collected. Next, NCBI’s BLASTCLUST^50^ with the local sequence identity at 25% (-S 25) was performed on the union of this set and the PDB972 dataset. The independent dataset was then created by selecting one sequence in each cluster that includes no chains from the PDB972 dataset. Meanwhile, similar constraints for each chain with length ≥ 60, resolution ≤ 2.0 Å and R-factor ≤ 0.2 are also satisfied. As a result, this set, called PDB3225, is composed of 3225 chains with a local sequence identity of 25% with each other and also with the sequences from the PDB972 dataset.

Moreover, we created another independent dataset extracted from MoDEL^51^ (Molecular Dynamics Extended Library), which is a database of protein trajectories obtained by means of state-of-art atomistic molecular dynamics simulations in near-physiological conditions. The aim of this independent dataset is to ascertain whether the proposed model can consistently perform well evaluated on MD-derived B-factors. First, the trajectories of MD simulation for Cα atoms of proteins were downloaded from the MoDEL database by taking the first simulation if there were multiple molecular simulations for the same protein. Second, BLASTCLUST^50^ with the local sequence identity at 25% (-S 25) was performed on the union of the proteins in MoDEL and the PDB927 dataset. Similarly as the procedure for collecting the PDB3225 dataset, an independent dataset, called MoDEL136, from MoDEL database was created and was finally composed of 136 chains with a local sequence identity of 25% with each other and with the sequences from the PDB972 dataset.

The PDB IDs of all protein chains in the PDB365, PDB607, PDB3225 and MoDEL136 datasets are listed in Tables S1, S2, S3 and S4 in Supplementary Information, respectively.

### Calculation of normalized B-factors and relative solvent accessibility

The experimental parameter, B-factor of an atom, is proportional to the isotropic mean square atomic displacement, i.e., defined as 8π^2^ <*u* ^2^> averaged over the lattice. Due to the fact that B-factor values are influenced by the experimental resolution, the refinement procedures and the crystal contacts, they are generally normalized for practical use between structures. Similarly as our previous study^14^, the B-factor values of *C*_*α*_ atoms for each protein chain were transformed using *B*’ = (*B* − *B*_*ave*_)/σ, where *B* is the actual B-factor value, *B*_*ave*_ is the mean actual B-factor in a given protein chain, and σ is the estimated standard deviation of actual B-factor values for all of the *C*_*α*_ atoms in a given protein chain.

For each protein in the MoDEL136 dataset, the *C*_*α*_ atoms’ 5001–10000 trajectories were downloaded from the MoDEL database. Then, the MD-derived B-factor of a residue in a protein was computed as:

1 $${{\rm{B}}}_{MD}=\frac{1}{J}\sum _{k=1}^{J}|{{\boldsymbol{r}}}_{k}-{{\boldsymbol{r}}}_{ave}{|}^{2}=\frac{1}{J}\sum _{k=1}^{J}[{({x}_{k}-{x}_{ave})}^{2}+{({y}_{k}-{y}_{ave})}^{2}+{({z}_{k}-{z}_{ave})}^{2}]$$

where *J* (equal to 5,000) is the total number of MD trajectories for a protein, ${{\boldsymbol{r}}}_{ave}=({x}_{ave},{y}_{ave},{z}_{ave})$ is the residue’s coordinate in the average structure of all trajectories, and ${{\boldsymbol{r}}}_{k}=({x}_{k},{y}_{k},{z}_{k})$ is the residue’s coordinate in the *k*th trajectory. The MD-derived B**’**-factors for further performance evaluation are also calculated using the same normalization as the experimental B-factors.

The actual ASA values in the three datasets PDB972, PDB3225 and MoDEL136 were computed with the DSSP program^52^. Following the work in (Dor and Zhou, 2007)^53^, RSA was computed by the ASA of a residue normalized by the ASA of this residue in its extended tripeptide (Ala-X-Ala) conformation^47^.

### Gaussian network model (GNM) and parameter-free GNM (pfGNM)

GNM describes each protein as an elastic network, where the springs linking the nodes denote the interactions between the residue pairs located within the distance cutoff *R*_*C*_^24^. Given that the springs are harmonic and the residue fluctuations are isotropic and Gaussian, the network potential of *N* nodes (residues) in a protein structure is

2 $${V}_{GNM}=\frac{\gamma }{2}\sum {\,}_{i,j}^{{\rm{N}}}{{\rm{\Gamma }}}_{ij}({{\bf{R}}}_{ij}-{{\bf{R}}}_{ij}^{0}{)}^{2}$$

where **R** ^0^_*ij*_ and **R**_*ij*_ are original and instantaneous distance vectors between residues *i* and *j*, respectively, γ is a constant of the force which is assumed to be uniform for all of the springs, and **Γ** = (Γ_*ij*_) is the Kirchhoff matrix defined as follows:

3 $${{\rm{\Gamma }}}_{ij}=\{\begin{array}{cc}-1, & {\rm{i}}{\rm{f}}\,i\ne j\,{\rm{a}}{\rm{n}}{\rm{d}}\,{{\rm{R}}}_{ij}^{0}\le {{\rm{R}}}_{{\rm{C}}}\\ 0, & {\rm{i}}{\rm{f}}\,i\ne j\,{\rm{a}}{\rm{n}}{\rm{d}}\,{{\rm{R}}}_{ij}^{0} > {{\rm{R}}}_{{\rm{C}}}\\ -\sum _{j:j\ne i}{{\rm{\Gamma }}}_{ij}, & {\rm{i}}{\rm{f}}\,i=j\end{array}$$

where *R* ^0^_*ij*_ is the original distance between residues *i* and *j* in the equilibrium state, and *R*_C_ is given as a distance cutoff. Thus, the mean square fluctuation of the *i* ^th^ residue is expressed as

4 $$\langle {\rm{\Delta }}{{\bf{R}}}_{i}^{2}\rangle =(3{k}_{{\rm{B}}}T/\gamma ){[{{\rm{\Gamma }}}^{-1}]}_{ii}$$

where *T* is the temperature and *k*_*B*_ is the Boltzmann constant. The cross-correlation map is then given by

5 $$\langle {\rm{\Delta }}{{\bf{R}}}_{i}\cdot {\rm{\Delta }}{{\bf{R}}}_{j}\rangle =(3{k}_{{\rm{B}}}T/\gamma ){[{{\rm{\Gamma }}}^{-1}]}_{ij}$$

which represents the mean correlations among residue fluctuations. Moreover, a parameter-free GNM (pfGNM), which substitutes for the distance cutoff by introducing a more physical definition of inverse power dependence between the residue-residue interactions, was proposed by Yang *et al*.^45^. The elements of the Kirchhoff matrix in pfGNM, are defined as

6 $${{\rm{\Gamma }}}_{ij}^{pf}=\{\begin{array}{cc}{({{\rm{R}}}_{ij}^{0})}^{-2} & {\rm{i}}{\rm{f}}\,i\ne j\\ -\sum _{j:j\ne i}{{\rm{\Gamma }}}_{ij}^{pf} & {\rm{i}}{\rm{f}}\,\,i=j\end{array}$$

### RSA based parameter-free Gaussian network model

In this study, the information about relative solvent accessibility (RSA) of all residues in a given chain was embedded into the Kirchhoff matrix of the pfGNM method. Inspired by our previous work^14^ that showed the contribution of the local impact of RSA values to the residue flexibility, local RSA differences between two residues are added into the proposed model. This method is called RSA based parameter-free Gaussian network model (RpfGNM) and its Kirchhoff matrix is defined as

7 $${{\rm{\Gamma }}}_{ij}^{Rpf}=\{\begin{array}{cc}{({{\rm{R}}}_{ij}^{0})}^{-2}\exp (\sum _{k=-h}^{h}{w}_{k}{(rs{a}_{i+k}-rs{a}_{j+k})}^{2}+b)\, & {\rm{i}}{\rm{f}}\,i\ne j\\ -\sum _{j:j\ne i}{{\rm{\Gamma }}}_{ij}^{Rpf}\, & {\rm{i}}{\rm{f}}\,i=j\end{array}$$

where *rsa*_*i*_ is the RSA value of the *i*th residue, the sliding window includes 2*h* + 1 residues, where *h* = 0, 1, 2, …, and the weighs *w*_*k*_ and intercept *b* are undetermined parameters that were estimated using particle swarm optimization.

### Parameter estimation using particle swarm optimization

Particle Swarm Optimization (PSO) has been successfully applied in several areas such as image processing^54^, parameter optimization^55^, and Quantitative Structure-Activity Relationship (QSAR) modeling^56^. Each particle in PSO is randomly initialized at a position in a given search space. The position for a particle *i* is given by a vector *x*_*i*_ = (*x*_*i*1_, *x*_*i*2_, …, *x*_*iD*_), where *D* represents the dimensionality of the search space. Velocity of a given particle is represented by the vector *v*_*i*_ = (*v*_*i*1_, *v*_*i*2_, …, *v*_*iD*_). PSO is an iterative algorithm in which the best position of the *i* ^th^ particle in previous iteration *t* is denoted by *p*_*i*_ = (*p*_*i*1_, *p*_*i*2_, …, *p*_*iD*_), and the best particle among all particles in the population is represented as *p*_*g*_ = (*p*_*g*1_, *p*_*g*2_, *…*, *p*_*gD*_). The particle updates its velocity and position according to the following two equations,

8 $${v}_{id}^{t+1}=a{v}_{id}^{t}+{c}_{1}{r}_{1}({p}_{id}-{x}_{id}^{t})+{c}_{2}{r}_{2}({p}_{gd}-{x}_{id}^{t})$$

9 $${x}_{id}^{t+1}={x}_{id}^{t}+{v}_{id}^{t+1}$$

where *d* is the *d* ^th^ dimension of a particle, *a* is the inertia weight, *c*_1_ and *c*_2_ are two positive constants called learning factors, and *r*_1_ and *r*_2_ are randomly generated ranged from 0 to 1^57^.

It is impossible to directly compute the proposed GNM before the weighs *w*_*k*_ and the intercept *b* as shown in Equation (7) are determined. In this work, the random optimization algorithm PSO is utilized to estimate these undetermined parameters. Here, the dimensionality of a particle equals 2*h* + 2 (*i.e*. *D* = 2*h* + 2), where the position vector *x*_*i*_ = (*x*_*i*1_, *x*_*i*2_, …, *x*_*iD*_) represents a vector composed of 2*h* + 2 parameters *w*_*−h*_, *w*_*−*(*h−*1)_, …, *w*_*h*_, and *b* in Equation (7).

The Pearson correlation coefficient (CC) is usually used to evaluate the predictive performance for real-value predictions^15, 47, 58, 59^ as well as the residue flexibility expressed as B-factor^15^. The other commonly used criterion is the mean absolute error, but due to the normalization of the raw B-factor values, this measure cannot be used to evaluate the quality of the flexibility predictors. The CC is defined as

10 $$CC=\frac{{\sum }_{i=1}^{N}({x}_{i}-\bar{x})({y}_{i}-\bar{y})}{\sqrt{[{\sum }_{i=1}^{N}{({x}_{i}-\bar{x})}^{2}][{\sum }_{i=1}^{N}{({y}_{i}-\bar{y})}^{2}]}}$$

where *x*_*i*_ and *y*_*i*_ are the observed B’-factor and the predicted B’-factor, respectively, for the *i* ^th^ residue in the sequence. If CC is close to 1, then {*x*_*i*_} and {*y*_*i*_} are fully correlated. If CC is close to 0 then the two variables are not correlated, and in the case when CC is close to −1 then the variables are anticorrelated. The absolute CC values quantify the degree of the correlation.

Similarly as in our previous work^14^, the correlation is measured at the protein chain level. The CC value is computed for each chain separately and next these values are averaged to compute the correlation over a given dataset. We use the term average correlation coefficient (ACC) to refer to the CC at the chain level.

To estimate the undetermined parameters in Equation (7) by using PSO, we need a fitness function to assess the performance of each particle. The ACC derived from the PDB365 dataset was used to define the fitness function for a particle. The parameters of the PSO-based optimizer were set as follows: the inertia weight *a* = 0.8, the learning factors *c*_*1*_ = 2 and *c*_*2*_ = 2, the population size of particles *NP* = 10, and the maximum number of iterations Iter = 20.

## Results

We estimated the undetermined parameters in the proposed RSA-based pfGNM based on the PDB365 dataset by using PSO. We then tested the proposed method on two independent sets PDB607 and PDB3225 by using the model learned from PDB365.

### Determination of the RSA-based parameter-free Gaussian network model

**Figure 1:**
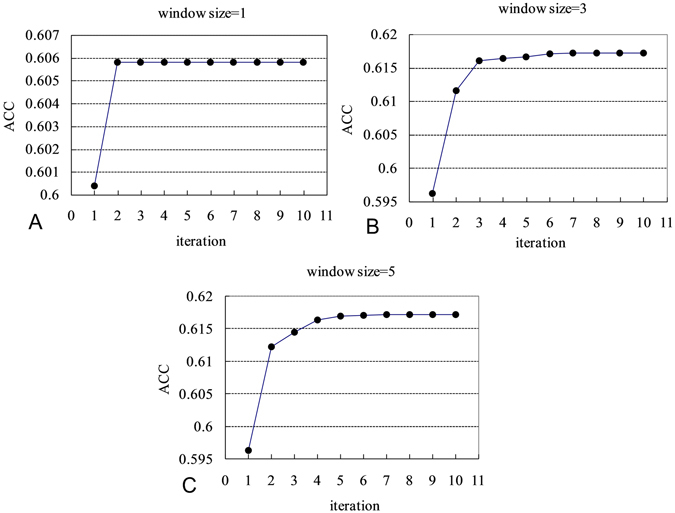
Plots of the ACC values with the increased iteration number resulted in the procedures for the PSO-based parameter estimations. Panels (A), (B) and (C) show the cases with sliding window sizes of 1, 3 and 5, respectively.

Figure 1 shows the plot of the ACC values with increased iteration number in the PSO-based optimization procedures for three cases of varying sliding window sizes (*h* = 0, 1, 2). We can find that the plots of two cases with window sizes of 3 and 5 are competitive and the highest ACC values are all around 0.617. We also performed optimization procedures for the cases with window sizes of 7 and 9, as a result, showing rather similar outcomes (not shown in Fig. 1) when compared with the case with a window size of 5. However, the highest ACC value for the case with a window size of 1 was achieved at only 0.606. Thus, a window size of 3 was selected and the number of parameters in the RSA-based pfGNM was consequently determined.

Moreover, for the case with a window size of 3 based on the PDB365 dataset, the weighs *w*_*−*1_, *w*_0_ and *w*_1_ in Equation (7) are estimated as −0.3589, −0.5061 and −0.4571, respectively, and the intercept *b* is −1.1003. Therefore, the Kirchhoff matrix of RpfGNM is determined as follows:

11 $${{\rm{\Gamma }}}_{ij}^{Rpf}=\{\begin{array}{ll}{R}_{ij}^{-2}\exp (-0.3589{(rs{a}_{i-1}-rs{a}_{j-1})}^{2}-0.5061{(rs{a}_{i}-rs{a}_{j})}^{2}\\ -0.4571{(rs{a}_{i+1}-rs{a}_{j+1})}^{2}-1.1003)\, & {\rm{i}}{\rm{f}}\,i\ne j\\ -\sum _{j:j\ne i}{{\rm{\Gamma }}}_{ij}^{Rpf} & {\rm{i}}{\rm{f}}\,i=j\end{array}$$

### Comparison of the RSA-based pfGNM with GNM and pfGNM

Table 1 shows the ACC values between the actual B’-factors and the predicted B’-factors by three GNM-type methods (i.e. the classical GNM, the parameter-free GNM and the proposed RSA-based pfGNM), three CN-type methods (i.e. the contact number, the weighted contact number (WCN), and the RSA combined WCN), and two RSA-type methods (i.e. RSA and DsspRSA9). Besides, the corresponding standard deviations of ACC values over the dataset are also reported. A distance cutoff of 8 Ǻ was used for the classical GNM, and the Kirchhoff matrices expressed in Equation (6) and Equation (11) were utilized for pfGNM and RpfGNM, respectively. The outputs of CN, WCN and RWCN are actually equal to the absolute diagonal elements of the Kirchhoff matrices of GNM, pfGNM and RpfGNM, respectively. The ACC between RSA values and native B-factors and the ACC of DsspRSA9 for prediction of B-factor are also reported. As shown in Table 1, the proposed RpfGNM achieved the best B-factor prediction performance of all considered methods except DsspRSA9. The ACC of the proposed RpfGNM is larger than those achieved by GNM and pfGNM for all three datasets PDB365, PDB607 and PDB3225. Meanwhile, the standard deviations achieved by RpfGNM were smaller when compared with GNM and pfGNM. Similarly, the CN-type RWCN consistently has higher ACC value than those of CN and WCN. In addition, GNM-type models outperformed their corresponding CN-type models, which implies that the GNM-type method can provides not only more information on protein dynamics such as correlated motion but also better predictions of the native B-factors. More importantly, the increase of ACC values on two independent datasets PDB607 and PDB3225 for RpfGNM are consistent with that was achieved based on the training dataset PDB365.

**Table 1:** The average correlation coefficients (ACCs) between the actual B’-factors and the predicted B’-factors computed by the GNM, pfGNM, RpfGNM, CN, WCN, RWCN, RSA and DsspRSA9 methods.

Method		PDB365	PDB607	PDB3225
GNM-type method	GNM	0.536(±0.2035)	0.568(±0.1613)	0.581(±0.1702)
	pfGNM	0.596(±0.1698)	0.621(±0.1439)	0.633(±0.1400)
	RpfGNM	**0.617**(±0.1614)	**0.641**(±0.1297)	**0.651(**±**0.1295)**
CN-type method	CN	0.489(±0.1283)	0.485(±0.0942)	0.506(±0.1029)
	WCN	0.586(±0.1408)	0.609(±0.1271)	0.616(±0.1196)
	RWCN	0.607(±0.1299)	0.626(±0.1176)	0.631(±0.1112)
RSA-type method	RSA	0.522(±0.10133)	0.524(±0.0898)	0.523(±0.0896)
	DsspRSA9	**0.655**(±1262)	**0.664**(±0.1054)	**0.659**(±0.1058)

*Note*: The computations were based on three datasets PDB365, PDB607 and PDB3225. The values in parentheses represent the standard deviations of ACC values.

**Figure 2:**
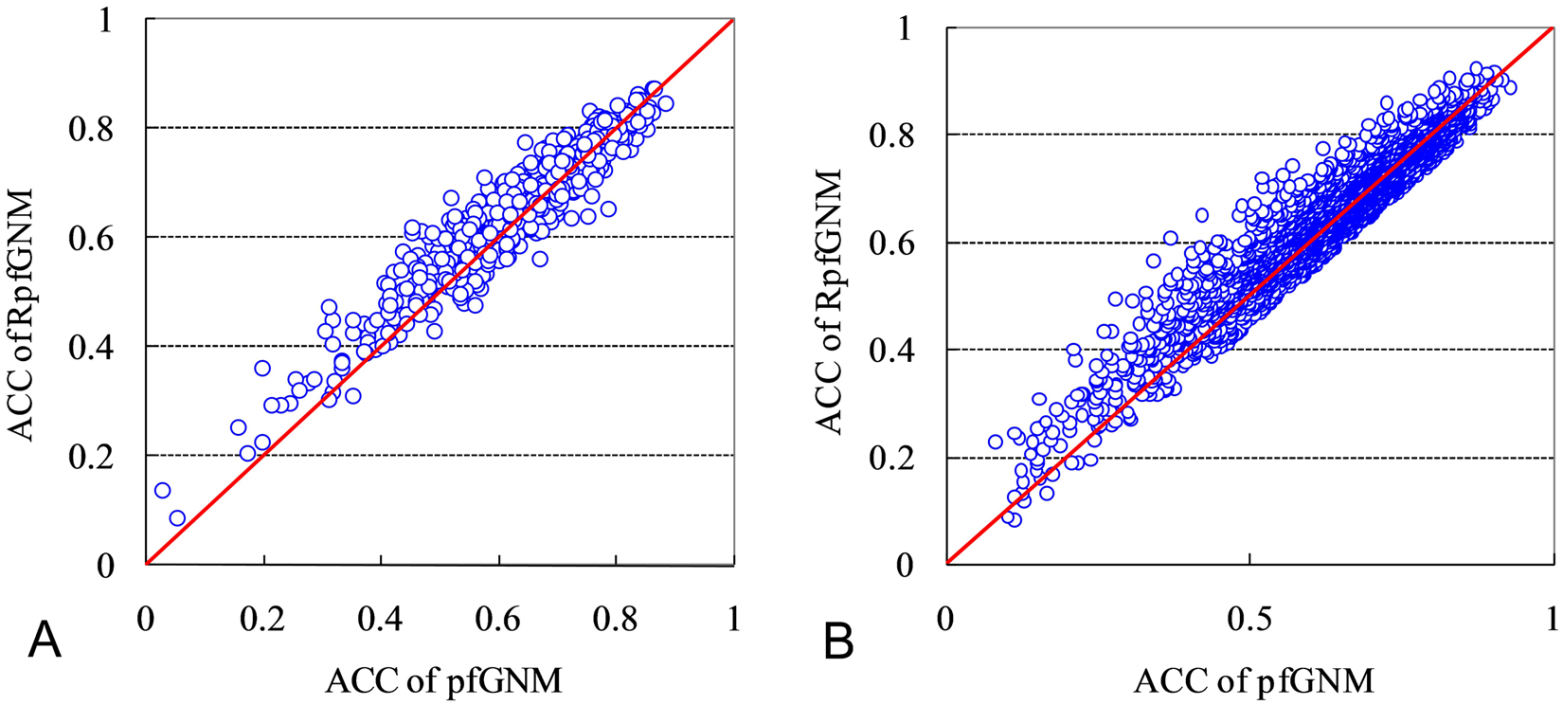
Comparison of the ACC values at the chain level between the pfGNM and the proposed RpfGNM based on blind tests on the PDB607 (panel A) and PDB3225 (panel B) datasets.

Figure 2 directly compares results for individual proteins between the pfGNM and the proposed RpfGNM based on the ACC values obtained on the datasets PDB607 (panel A) and PDB3225 (panel B). We performed paired t-tests to compare pairs of ACC values for the same sequences predicted by the pfGNM and the proposed RpfGNM at the significance level of 0.05. The *p*-values for both blind tests on the PDB607 and PDB3225 datasets are below 0.0001, which suggests that the differences between the pfGNM and the proposed RpfGNM are statistically significant. Furthermore, the (proposed) RpfGNM provides higher ACC values for a majority of the predicted sequences when compared with the pfGNM, i.e., most of the points are located above the diagonal red line. More specifically, in the case of the PDB607 dataset, 412 out of 607 proteins have higher ACC values for the proposed RpfGNM. Similar findings are true for the PDB3225 dataset where 2006 out of 3225 proteins are above the diagonal.

Table 2 analyzes the predictive quality of GNM, pfGNM and RpfGNM validated on several protein subsets according to varying sequence lengths. The lengths of protein chains were divided into seven intervals, as shown in the table. It lists the ACC values of GNM, pfGNM and RpfGNM for the PDB3225 dataset. First of all, it can be observed that the proposed RpfGNM consistently outperformed GNM and pfGNM for each protein subset according to different length intervals. The standard deviations of ACC values over each protein subset were consistently reduced by RpfGNM when compared with GNM and pfGNM. Secondly, the increment of ACC values for RpfGNM when compared with pfGNM range from 0.009 to 0.024. The proposed method tends to benefit the improvement of ACC values for short sequences, especially for the chains with length less than 200. Most likely this is primarily attributed to the distribution of RSA values over the whole protein structure. We computed the ratio of residues with exactly zero RSA values, which indicates these residues are completely inaccessible to solvents, to the entire residues for each chain. Similarly, the ratio of residues with RSA value <= 25% that was widely used to define buried and exposed residues was also calculated. It has been shown in Table 2 that the protein chains with shorter lengths also tend to have lower mean ratios of residues with zero RSA values or less than 25%. Actually, the computation for an element of the Kirchhoff matrix proposed in Equation (11) is dependent on the difference between RSA values of two residues. If two residues have RSA values that are equal to exactly zero or less than 25%, the difference of these two RSA values will consequently also be equal to exactly zero or relatively small, which results in no, or weak discrimination about solvent exposure information between two residues.

**Table 2:** The ACC values of GNM, pfGNM and RpfGNM calculated on subsets of the PDB3225 dataset according to varying sequence lengths with step size of 100.

Range of length (L)	No. of proteins	GNM	pfGNM	RpfGNM	Mean ratio of residues with zero RSA values	Mean ratio of residues with RSA value <=25%
L < 100	338	0.574(±0.2186)	0.628(±0.1857)	**0.652**(±0.1747)	8.18%	42.21%
100 <= L < 200	1010	0.574(±0.1828)	0.630(±0.1461)	**0.650**(±0.1367)	10.83%	49.47%
200 <= L < 300	819	0.580(±0.1573)	0.631(±0.1264)	**0.647**(±0.1177)	14.00%	55.87%
300 <= L < 400	592	0.582(±0.1466)	0.631(±0.1299)	**0.649**(±0.1167)	15.45%	59.22%
400 <= L < 500	261	0.588(±0.1545)	0.643(±0.1210)	**0.656**(±0.1113)	15.44%	60.46%
500 <= L < 600	109	0.615(±0.1407)	0.666(±0.1234)	**0.677**(±0.1054)	15.88%	62.72%
L >= 600	96	0.612(±0.1393)	0.653(±0.1138)	**0.662**(±0.0979)	15.96%	63.29%

*Note*: The values in parentheses represent the standard deviations of ACC values over the corresponding protein subset. The mean ratios of buried residues based on RSA cutoffs of zero and 25% are also included.

It can be observed in Tables 1 and 2 that the improvement on B-factor predictions is relatively marginal when the proposed RpfGNM is compared to the pfGNM. This may be due to the fact that the Kirchhoff matrix of the proposed RpfGNM was established by embedding the RSA information of residue pairs into the pfGNM. The diagonal element for a residue in Kirchhoff matrix of the pfGNM model is actually the weighted contact number (WCN)^22^. We examined the relation between RSA and WCN by calculating the average correlation coefficient (ACC). The ACC values between RSA and WCN based on the datasets PDB365 and PDB3225 are 0.739 and 0.728, respectively. As expected, the improvement on X-ray B-factor predictions of the RpfGNM is not so remarkable when compared with the pfGNM. However, RSA values are still able to provide complementary contribution to the enhancement of the B-factor predictions according to our computational experiments although they are correlated with WCN values.

Moreover, we here assumed that a residue is exposed (*e*) if its RSA value is larger than the cutoff of 25%, and otherwise it is defined as buried (*b*). Similarly as our previous work^14^, we investigated the exposure patterns of tripeptides in which one central residue with exposure state *x* (*e* or *b*) may have two buried (*bxb*), two exposed (*exe*), or one buried and one exposed (*exb* and *bxe*) adjacent neighbors. By computing the mean B’-factors of the central residues for six possible tripeptide exposure patterns as shown in Table 3, we found that the exposed residues with two buried adjacent neighbors (i.e., *beb* pattern) have lower mean B’-factor value than the buried residues with two exposed adjacent neighbors (i.e., *ebe* pattern). Specifically, the mean B’-factor value is −0.042 for the central residues with *beb* pattern, while it is 0.119 for *ebe* pattern, based on the PDB 3225 dataset. This observation is in good agreement with our earlier finding^14^, implying that two buried neighbors may strongly influence the flexibility of the central residue making it more rigid than the buried residue which is flanked by two exposed residues. This may also serve as a reason why the RSA information can improve the predictions of the residue flexibility.

**Table 3:** Mean B’-factor values for the six tripeptide exposure patterns with RSA cutoff of 25% based on the PDB3225 dataset, where the corresponding standard deviations are also included in parentheses.

Exposure of the central residue	Tripeptide exposure pattern	No. of residues	Mean B’-factor
Buried	bbb	218956	−0.621(±0.4589)
	bbe/ebb	178245	−0.239(±0.6553)
	ebe	75006	0.119(±0.8629)
Exposed	beb	74334	−0.042(±0.7297)
	bee/eeb	178292	0.379(±0.9878)
	eee	97142	0.902(±1.3104)

In addition, we compared the mean values of actual, pfGNM-predicted and RpfGNM-predicted B’-factors for buried and exposed residues, which were defined using RSA cutoff of 25% based on the PDB3225 dataset. As shown in Table 4, both mean pfGNM-predicted and RpfGNM-predicted B’-factors are lower than the mean actual B’-factor for buried residues, while they are larger than the mean actual B’-factor for exposed residues. However, the mean RpfGNM-predicted B’-factor is larger (lower) than that of pfGNM for buried (exposed) residues. This implies that the proposed RpfGNM provide much closer predictions to the actual B’-factor values when compared with pfGNM. Especially for exposed residues, RpfGNM seems to repress the over evaluated fluctuations of surface residues by pfGNM, which may confirm the fact that the estimated parameters for RSA terms in the RpfGNM (see Equation (11)) turn down the interactions between the surface residues and buried residues.

**Table 4:** Mean values of the actual, pfGNM-predicted and RpfGNM-predicted B’-factors for buried and exposed residues defined using RSA cutoff of 25% based on the PDB3225 dataset, where the corresponding standard deviations are also included in parentheses.

Exposure of residues	Mean actual B’-factor	Mean pfGNM-predicted B’-factor	Mean RpfGNM-predicted B’-factor
Buried	−0.358(±0.6734)	−0.500(±0.6473)	−0.484(±0.5883)
Exposed	0.476(±1.1527)	0.664(±0.9995)	0.643(±1.0695)

In contrast to CN-type and RSA-type methods listed in Table 1, the proposed RpfGNM has also the ability to generate the cross-correlations of residue fluctuations and to describe the correlated motions of residues in a given protein. Similarly as in our previous study^16^, we computed the ACCs of the cross-correlations of residue fluctuations for all pairs of considered methods including GNM, pfGNM and RpfGNM on the PDB3225 dataset; see Table 5. The ACC value between GNM and RpfGNM (or between GNM and pfGNM) is around 0.6 on the PDB3225 dataset when a cutoff of 8 Ǻ for GNM was used, which indicates that both pfGNM and RpfGNM can generate similar cross-correlation matrices with GNM. However, this ACC value is significantly lower than that was reported in our previous work^16^, where a larger cutoff of 12 Ǻ for GNM was adopted. Given a distance cutoff of 12 Ǻ for GNM, the ACC value between the cross-correlation matrices by GNM and that by RpfGNM (or GNM and pfGNM) was improved to be around 0.8 which was also shown in Table 4. It is reasonable to assume that when longer residue-residue contacts along with the increase of distance cutoff are added, the Kirchhoff matrices of GNM, pfGNM and RpfGNM become closer. On the other hand, the ACC value between pfGNM and RpfGNM is 0.995 on the PDB3225 dataset, indicating that the cross-correlation matrices generated by these two methods are very close.

**Table 5:** The ACCs between the cross-correlations of residue fluctuations by GNM, pfGNM and RpfGNM on the PDB3225 datasets.

Method	GNM	pfGNM	RpfGNM
GNM	1	0.599(±0.1134)/0.808(±0.1221)^a^	0.603(±0.1143)/0.814(±0.1217)^a^
pfGNM		1	0.995(±0.0036)
RpfGNM			1

^a^The left value means the distance cutoff used in GNM is 8 Ǻ, while the right value corresponds to the cutoff of 12 Ǻ used in GNM. The values in parentheses represent the standard deviations of ACC values over the corresponding dataset.

### Evaluation on MD-derived B-factors

Table 6 shows the ACC values between the actual B’-factors (or MD-derived B’-factors) and the predicted B’-factors computed by GNM-type, CN-type and RSA-type methods based on the MoDEL136 dataset. For GNM-type and CN-type methods, the ACC values evaluated on MD-derived B’-factor are higher than those assessed using the actual B’-factor on the same dataset MoDEL136. We also observed that the proposed RpfGNM optimized by using the actual B’-factors can still provide improved predictions that was evaluated on the MD-derived B’-factors when compared with pfGNM. It is consistent in a comparison of the RWCN method with WCN, although the ACC improvement of the RWCN method is small when compared with WCN. In contrast, RSA-type methods, including RSA and DsspRSA9, generate lower ACC values evaluated on MD-derived B’-factors when compared with the actual B’-factors.

**Table 6:** The average correlation coefficients (ACCs) between the actual B’-factors (or MD-derived B’-factors) and the predicted B’-factors computed by the GNM, pfGNM, RpfGNM, CN, WCN, RWCN, RSA and DsspRSA9 methods based on the MoDEL136 dataset.

Method type	Method	Actual B’-factor	MD-derived B’-factor
GNM-type method	GNM	0.568(±0.2110)	0.657(±0.1446)
	pfGNM	0.611(±0.1785)	0.664(±0.1291)
	**RpfGNM**	**0.626**(±0.1743)	**0.678**(±0.1264)
CN-type method	CN	0.484(±0.1212)	0.465(±0.0896)
	WCN	0.587(±0.1432)	0.575(±0.1043)
	RWCN	0.602(±0.1345)	0.573(±0.1062)
RSA-type method	RSA	0.481(±0.1134)	0.466(±0.0839)
	DsspRSA9	0.617(±0.1506)	0.600(±0.0945)

*Note*: The values in parentheses represent the standard deviations of ACC values over the corresponding dataset.

Moreover, we computed ACC values between the cross-correlations of residue fluctuations by pfGNM, RpfGNM and MD based on the MoDEL136 dataset. The cross-correlation map of MD was actually calculated as the covariance matrix of all MD trajectories for a protein^60^. As a result, the ACC value between the cross-correlation maps of MD and RpfGNM is 0.404 ± 0.0844, which is higher than that of 0.386 ± 0.0811, between MD and pfGNM. It was surprising that the ACC values between cross-correlation maps of GNM and MD are 0.507 ± 0.1097 and 0.478 ± 0.0997 when the distance cutoffs in GNM are 8 Å and 12 Å, respectively. However, the improvement is consistent while RpfGNM is compared to pfGNM, although all of these ACC values between GNM-type methods and MD are relatively low. Note that the residue fluctuations in GNM-type methods are assumed to be isotropic and the potentials are harmonic. By contrast, MD simulation adopts anharmonic potentials and there is no isotropic hypothesis for residue fluctuations in MD simulation. This may be the key points that result in low similarity between the cross-correlation maps of GNM-type models and MD simulations.

The improvement of ACC values on the MoDEL136 dataset shown in Table 6 by comparing RpfGNM with pfGNM is achieved by optimizing the RpfGNM model based on the PDB365 dataset. It will be interesting to investigate the parameter optimization of RpfGNM based on the MoDEL136 dataset that may further increase the MD-derived B-factor predictions. After the same optimization of RpfGNM by utilizing PSO based on the MoDEL136 dataset, the highest ACC value associated with the global best particle in PSO algorithm is 0.681. It is really a marginal increase (only ~0.003) when compared with the ACC value of 0.678 listed in Table 6, which is achieved by the MD-derived B’-factor predictions of RpfGNM evaluated on the MoDEL136 dataset. From the view of B’-factor prediction, the overall performance of RpfGNM gained by the optimizations using X-ray B-factors and MD-derived B-factors is very close. The proposed RpfGNM optimized on X-ray B-factors can be applied to the prediction of MD-derived B-factors with ACC improvement to some extent.

### Case study

We further investigated the outputs of GNM, pfGNM and RpfGNM in context of case study by observing one protein: cytochrome c3 from *DesulfoVibrio desulfuricans*(PDB ID: 1AQE)^61^. Cytochrome c3 are extensively studied proteins which play a central role in energy transduction by the transfer of electrons and protons from hydrogenase^62^. We computed the CC values between the predicted B-factors by three considered methods (GNM, pfGNM, RpfGNM) and the actual/MD-derived B-factors of Cytochrome c3 using a PDB structure (PDB ID: 1AQEA) that was included in the MoDEL136 dataset; see Table 7. It can be observed that the improvements of the proposed RpfGNM when compared with pfGNM for the B-factor prediction are consistently achieved by CC values of 0.026 in case of actual B-factors and 0.036 for MD-derived B-factors.

**Table 7:** The CC values for cytochrome c3 between the actual/MD-derived B-factors and the predicted B-factors by GNM, pfGNM and RpfGNM.

Method	CC against the actual B-factors	CC against the MD-derived B-factors
GNM	0.460	0.440
pfGNM	0.484	0.676
RpfGNM	0.510	0.712

*Note*: The actual B-factors of cytochrome c3 are extracted from a PDB structure with PDB id 1AQEA. The distance cutoff used in GNM is 8 Ǻ.

**Figure 3:**
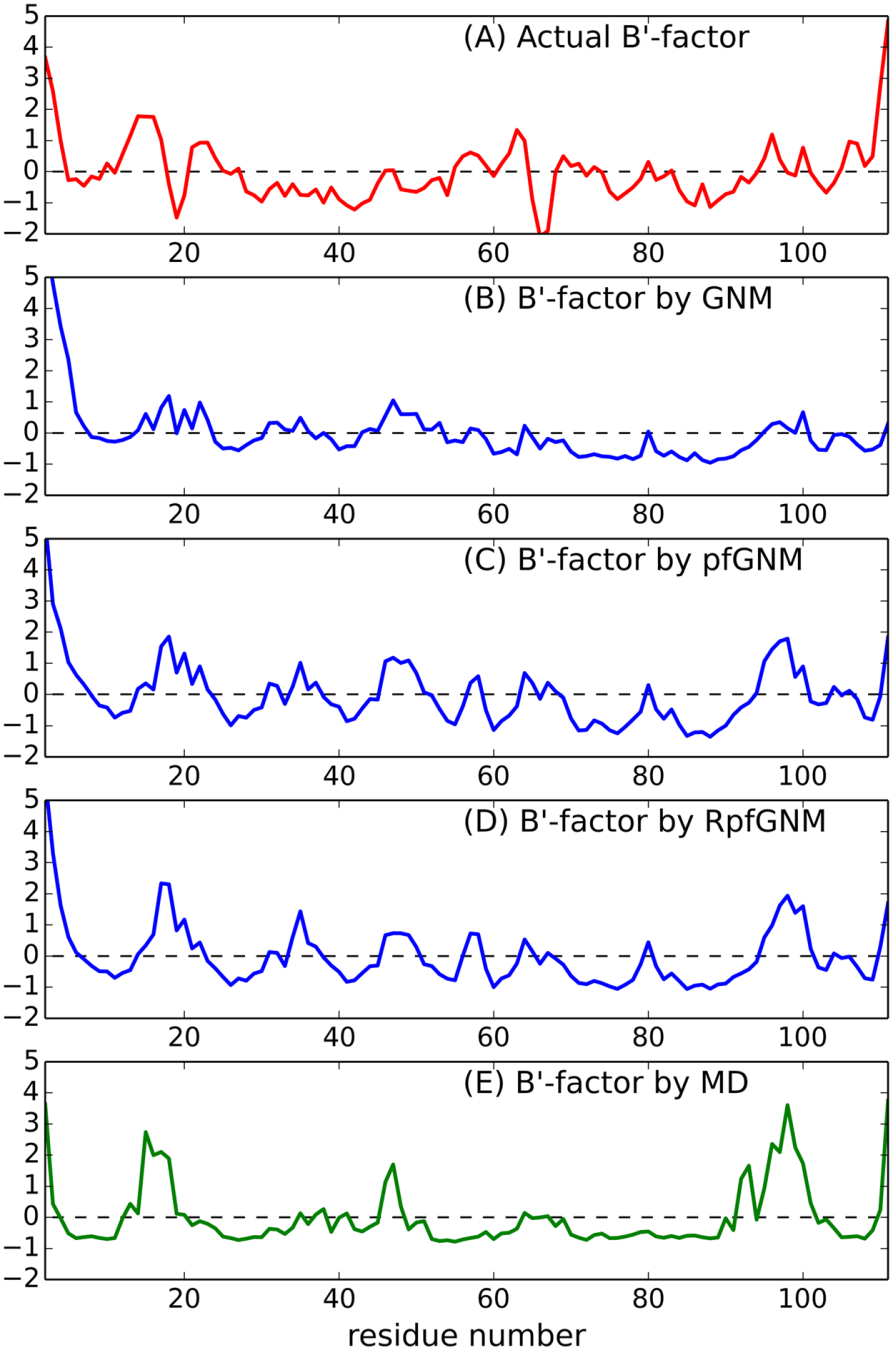
Plots of the actual B’-factor profile (panel A) and the B’-factor profiles predicted with GNM (panel B), pfGNM (panel C), RpfGNM (panel D) and MD simulation (panel E) for cytochrome c3 (PDB: 1AQEA).

**Figure 4:**
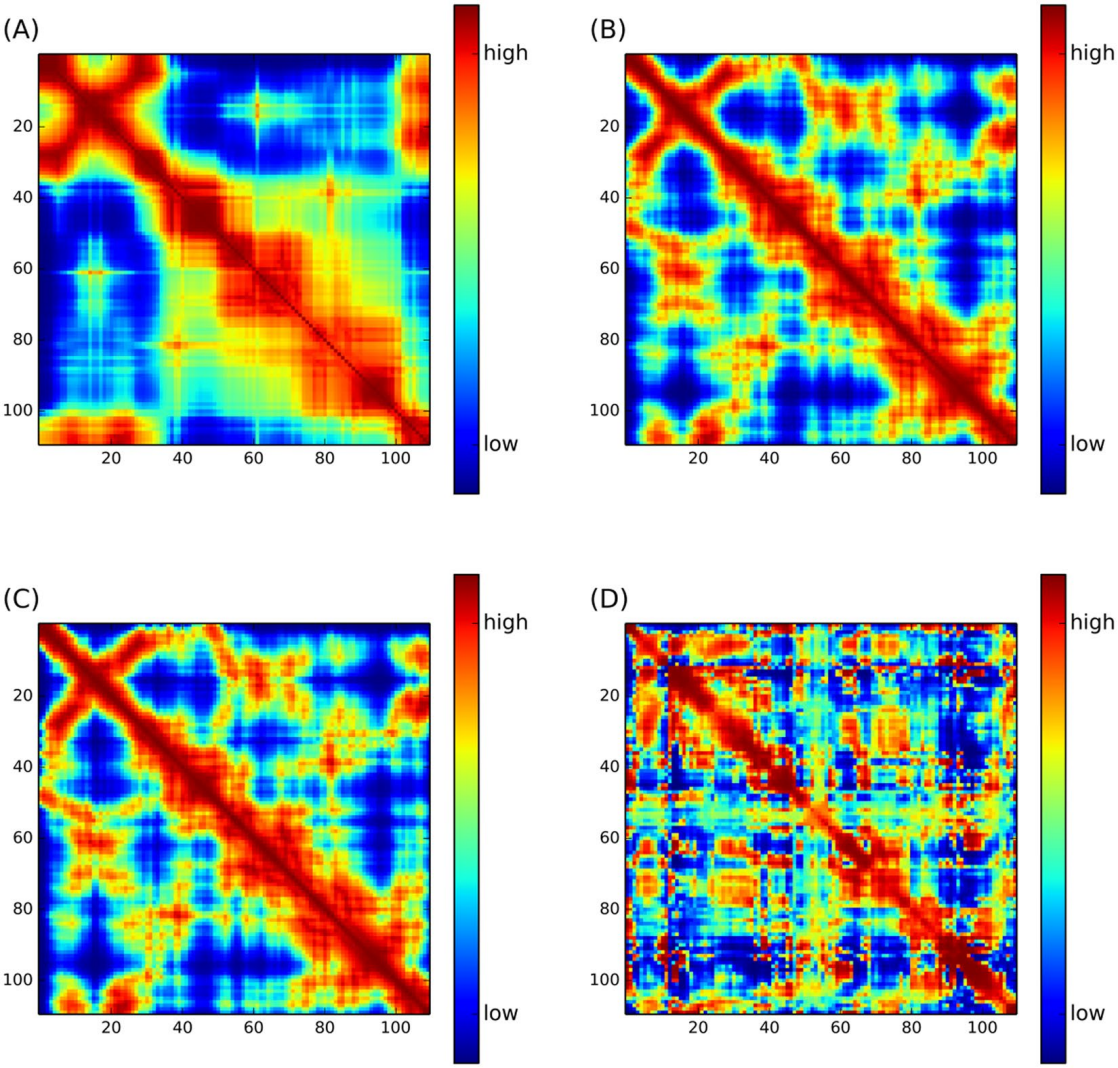
The maps of cross-correlations of residue fluctuations for cytochrome c3 (PDB:1AQEA) computed with (**A**) GNM, (**B**) pfGNM, (**C**) RpfGNM, and (**D**) MD.

Figure 3 plots and compares the actual B’-factor profile of cytochrome c3 (PDB: 1AQEA) as well as the predicted B’-factor by GNM, pfGNM, RpfGNM and MD. Figure 4 shows the cross-correlations of residue fluctuations of cytochrome c3 generated by GNM, pfGNM, RpfGNM and MD, respectively. It can be easily observed that the majority of peaks in the actual B’-factor profile are correctly identified by four considered computational methods, i.e. GNM, pfGNM, RpfGNM and MD, as shown in Fig. 3. As shown in Fig. 3, there are two low peaks, i.e. GLU17-PRO21 and LYS64-GLU68, in the actual B’-factor profile (panel (A) in Fig. 3), but they are both absent in the four B’-factor profiles derived by GNM, pfGNM, RpfGNM and MD, respectively. This seems to be the most obvious discrepancy between the benchmarks using the actual B’-factor profile and the MD-derived B’-factor profile. Nevertheless, all of the predicted B’-factor profiles generated by GNM, pfGNM and RpfGNM do not show these two low peak, which results in higher ACC values between the B’-factors predicted by pfGNM and RpfGNM and the benchmark against MD-derived B’-factors when compared with the actual B’-factors. Moreover, both the B’-factor profile and the map of cross-correlations of residue fluctuations for cytochrome c3 generated by RpfGNM are overall very close to those by pfGNM. There is a fact that the high peak around GLU17 in the B’-factor profile of RpfGNM is relatively narrower than those of GNM and pfGNM, which may be the main contribution to the ACC improvement for RpfGNM when compared with pfGNM for both actual B-factors and MD-derived B-factors as benchmark.

## Discussion

Gaussian network model (GNM), a very simple coarse-grained model, has been widely applied to describe and analyze protein dynamics and functions. Designing new variations of the classical GNM is the way to reduce the gap between the quality of the dynamics of all-atom models and that of coarse-grained models. The vital computation of the GNM is to define the Kirchhoff matrix. We proposed a new Kirchhoff matrix based on the previous parameter-free GNM^45^ by combining the information about local relative solvent accessibility between two residues. The undetermined parameters in the new Kirchhoff matrix are estimated by using PSO. The computational experiments demonstrated that the proposed model, RpfGNM, achieved an increment in ACC value by around 0.02 when compared with the parameter-free GNM based on one training dataset and two independent datasets. Therefore, our empirical results showed that extra information, such as the distance and the difference of RSA values between two residues, is useful to improve the flexibility modeling of GNM that is expressed using B-factor.

The usage of RSA is motivated by the high-quality of the structure-based method DsspRSA9 that used to investigate the relationship between the residue flexibility measured using B-factor and the local solvent accessibility in our previous work^14^. DsspRSA9 provided better predictions of B-factors when compared with the classical GNM and the parameter-free GNM^14, 16^, which was also confirmed by the results derived from the independent dataset PDB3225. In this study, the proposed RpfGNM improved the B-factor predictions when compared with pfGNM and obtained quality closer to DsspRSA9. The finding implies that the flexibility modeling of GNM may be further improved by combining other structural properties of proteins, such as secondary structure besides just solvent accessibility.

Recently, several models tested against experimental X-ray B-factors as benchmark were developed. For example, a model termed translation, libration and screw (TLS), proposed by Soheilifard *et al*.^63^, obtained ACCs greater than 0.8 on certain datasets by taking the rigid-body motions into account. Li and Brüschweiler^64^ proposed local contact models for predicting X-ray B-factors achieved by CC values over 0.70. Song and Jernigan^46^ proposed a model, called vGNM, achieved by ACC value of 0.81, which included both the contribution of the rigid body motions and the effect of crystal packing by allowing the amplitudes of the low frequency modes of GNM to be variable. Kundu *et al*.^24^ improved the ACC value of GNM to 0.661 against the X-ray B-factors by incorporating the effect of neighboring molecules in the crystal. We can observed that the ACC improvement was very remarkable when the classical GNM^23^ was compared to the models like TSL and vGNM on B-factor predictions. By contrast, the ACC improvement of RpfGNM when compared with the classical GNM is relatively small. However, we note that these models are not comparable directly with the proposed RpfGNM. Nevertheless, three methods including TSL, LCM and vGNM cannot generate normal modes as well as cross-correlation maps which can be utilized for exploring protein intrinsic dynamics while the classical GNM or its variations constructed from Kirchhoff matrix can do. They were concentrated on fitting the crystal B-factors and aiming to achieve higher ACC values. Additionally, the fourth approach proposed by Kundu *et al*.^24^ embedded the information about the neighboring molecules into the classical GNM and then improved the performance of GNM against B-factors as benchmark, while our study focused on developing a variation of the parameter-free GNM that was proposed by Yang *et al*.^45^ without incorporating crystal neighbors. The proposed RpfGNM enhanced B-factor predictions and also retains the advantage that it can generate normal modes and cross-correlation maps which are useful for exploring protein intrinsic dynamics.

It has been recommended that molecular dynamics (MD) simulation may generate much more reliable outputs describing internal dynamics of proteins and MD-derived B-factors or covariance will be a better alternative as benchmark for optimizing model parameters^60^. However, MD simulations are often computationally prohibitive, especially when long time scales need to be taken into account^65^. We evaluated the proposed RpfGNM based on a relatively small dataset composed of 136 proteins that were collected from MoDEL database. The results suggested that the proposed RpfGNM showed the consistent improvement on ACC value when using MD-derived B-factors as benchmark. We believe that the proposed RpfGNM is able to achieve consistent improvement with a comparison to pfGNM if sufficient data about MD-derived B-factors are available as benchmark and the optimization is reperformed to determine the model parameters.

This work can be viewed as an alternative way to design novel variations of the classical GNMs. We shall make efforts in our future work to develop more variations of classical GNMs as well as the variations for another type of ENM, called anisotropic network model (ANM). The variations with better performance are promising for finding numerous applications in areas such as high-quality flexibility modeling for protein motions, conformational changes and protein functions.

## Electronic supplementary material

Supplementary Information
