# Supplementary material for: Gaussian network model can be enhanced by combining solvent accessibility in proteins
Source: Sci Rep. 2017 Aug 8;7:7486. doi: 10.1038/s41598-017-07677-9 (PMC5548781; doi:10.1038/s41598-017-07677-9)
Supplement: Supplementary file 1 — Supplementary Information [file 41598_2017_7677_MOESM1_ESM.doc]

Gaussian network model can be enhanced by combining solvent accessibility in proteins

Hua Zhang^1^*, Tao Jiang^2^, Guogen Shan^3^, Shiqi Xu^1^, Yujie Song^1^

^1^School of Computer and Information Engineering, Zhejiang Gongshang University, Hangzhou, Zhejiang, PR China 310018.

^2^School of Statistics and Mathematics, Zhejiang Gongshang University, Hangzhou, Zhejiang, PR China 310018.

^3^School of Community Health Sciences, University of Nevada Las Vegas, Las Vegas, NV 89154, USA

**Supplementary Information**

**Table S1.** A list of the PDB ids in the PDB365 dataset.

| 1A1IA  1A6MA  1AGQD  1AMMA  1AOCA  1APYA  1AW7A  1AY7B  1AYOA  1B3AA  1B6TA  1B8EA  1BGFA  1BHTA  1BXEA  1C48A  1C5EA  1C7KA  1C9OA  1CC8A  1CCWA  1CCZA  1CHDA  1CMBA  1CQYA  1CV8A  1CZ9A  1D0DA  1D4OA  1D7PM  1DBFA  1DG6A  1DGWX  1DJTA  1DUNA  1DUPA  1DY5A  1E7LA  1EB6A  1EH7A | 1EJBA  1EKGA  1EL4A  1ELKA  1EPFB  1EX2A  1EXRA  1EXTA  1F4NA  1F86A  1FK5A  1FLTX  1G1TA  1G2BA  1G3PA  1GBSA  1GCQC  1GCVB  1GD0A  1GK8I  1GMXA  1GNUA  1GUIA  1GWMA  1GXUA  1H2CA  1H4YA  1H6FB  1H6KX  1HDKA  1HFES  1HG7A  1HH8A  1HPIA  1HZ5B  1HZTA  1I1DD  1I2TA  1I4UA  1I6TA | 1I7QB  1I8OA  1IABA  1IDPA  1IFCA  1IIBA  1IPCA  1IQZA  1IV9A  1J0PA  1J2RA  1J34A  1J8BA  1JBEA  1JD5A  1JPCA  1JRLA  1K12A  1K6ZA  1KHIA  1KNLA  1KOEA  1KT7A  1KVEA  1KZKB  1L2HA  1L3KA  1L9LA  1LATB  1LK2B  1LKIA  1LKKA  1LNIB  1LOVA  1LQVB  1LTSA  1LWBA  1LY2A  1M2DA  1M4IB | 1M55A  1M9XC  1M9ZA  1MK0A  1MKAA  1MKKA  1MN8D  1MQKH  1N13B  1N7SA  1N7SC  1NKIA  1NOXA  1NQJA  1NU0A  1NWAA  1NWZA  1NYCA  1O7IA  1O7NB  1O83A  1O8XA  1OK0A  1OOHA  1OR7C  1P6OB  1PA7A  1PBJA  1PI1A  1PK6A  1PL3A  1PM1X  1PM4A  1PMHX  1POCA  1PSRB  1PT6B  1PVMB  1PYOC  1Q0NA | 1Q40B  1Q7LA  1Q7LB  1QB5D  1QF8B  1QFTA  1QGWB  1QIPA  1QKRB  1QR9A  1QW2A  1QX2A  1R29A  1R2QA  1R6JA  1R8SA  1RA9A  1RG8A  1RIEA  1RKIA  1RLID  1RROA  1RUTX  1RXQB  1RY9A  1RYAA  1S4KA  1S67L  1S7FA  1S7ZA  1S99A  1SAUA  1SJWA  1SMBA  1SQEB  1SVFA  1SWXA  1T92A  1TO2I  1TQGA | 1TT8A  1TU1A  1TU9A  1TUKA  1TXJA  1TXQB  1TZVA  1U07B  1U11B  1U69D  1U7IA  1U9DA  1UCDA  1UGHI  1UGNA  1UKUA  1UNNC  1UNQA  1UOWA  1UPGA  1USCA  1UW1A  1UWFA  1UXZA  1UZKA  1V5IB  1V6PA  1V70A  1VFYA  1VH5A  1VL9A  1VLSA  1VR7A  1VYIA  1VYKA  1VZIA  1W0HA  1W0NA  1W4RA  1W94A | 1WAPA  1WC2A  1WC3B  1WDCA  1WHIA  1WKQA  1WLDA  1WM3A  1WPNA  1WRIA  1WS8A  2V1TB  1WV3A  1WWCA  1WY1A  1WYXB  1X0JA  1X6IB  1X82A  1XEOA  1XERA  1XHDA  1XKPB  1XKPC  1XWWA  1Y2TA  1Y43B  1Y63A  1Y93A  1Y9WA  1YGTA  1YJ1C  3PMCA  1YN9A  1YO3A  1YPHC  1YPHE  1YPQB  1YRKA  1YTBA | 1YU8X  1YVIA  1Z1SA  1ZCEA  2R99A  1ZNDA  1ZZWA  2A50A  2A50B  2AB0A  2ACFB  2AD6B  2AENB  2AGYD  2AIBA  2APXA  2AQ6A  2ARPF  2AUWB  2AWGA  2AXWA  2B06A  2B58A  2B5HA  2B97A  2BEMA  2BF5A  2BKFA  2BOPA  2BRFA  2BSWA  2BT9A  2BW3B  2BWQA  2BZUA  2C0NA  2C2UA  2C9VA  2CARA  2CJTC | 2CK3G  2CKLA  2CKLB  2CVIA  2CWGA  2CXXC  2DBBB  2DKOB  2EXVC  2F01B  2F2QA  2F4MB  2FA8C  2FHAA  2FHZA  2FL7A  2FP7B  2FPEA  2FRGP  2FSAA  2FSRA  2FWGA  2FYGA  2FYQA  2G2WB  2G7OA  2GAGD  2GBAA  2GRHA  2GRRA  2GRRB  2GS5A  2GUDB  2H29A  2H88D  2HTSA  2IU1A  2IU5A  2LISA  2MHRA | 2TGIA  3CHBF  3VUBA  4LZTA  7FD1A |
| --- | --- | --- | --- | --- | --- | --- | --- | --- | --- |

**Table S2.** A list of the PDB ids in the PDB607 dataset.

| 1A53A  1A8DA  1A8IA  1A9XA  1ADEA  1ADOA  1AF7A  1AGJA  1AH7A  1AJSA  1AOPA  1ARBA  1B5QA  1B65A  1BF2A  1BGVA  1BIFA  1BSLB  1C0PA  1C1KA  1C7SA  1CB0A  1CCWB  1CFBA  1CQXA  1CRUB  1CSHA  1CVRA  1CZFA  1D8DA  1DC1B  1DDTA  1DFMA  1DJ0A  1DJEA  1DLJA  1DMRA  1DOZA  1DQAA  1DQZA  1DS1A  1DXRM  1E1HA  1E4CP  1E6PB  1E6UA  1E9EA  1E9GB  1EBLA  1ECFB  1EDGA  1EDQA  1EEOA  1EEXA  1EJDA  1EKXA  1EQCA  1ES9A  1ESGB  1EU8A  4UBPC | 1EUVA  1F1XA  1F20A  1F24A  1F8EA  1FCQA  1FEHA  1FIUA  1FKMA  1FN9A  1FO8A  1FP3A  1FS7A  1FSGC  1FUPA  1G60A  1G61A  1G66A  1G8AA  1G8KA  1G9GA  1GK9A  1GK9B  1GKPA  1GNLA  1GOFA  1GQIA  1GQYB  1GTED  1GUQA  1GVKB  1GWEA  1GX5A  1GXMB  1H16A  1H1IB  1H32A  1H4GB  1H6KC  1HBNA  1HBNB  1HBNC  1HDOA  1HF8A  1HP1A  1HQSA  1HS6A  1HT6A  1HYOB  1HZ4A  1I19A  1I1NA  1I9ZA  1IC6A  1ITUA  1IU8A  1IUQA  1IV8A  1IXBA  1J0HA  7AHLB | 1J79B  1JAKA  1JDWA  1JEVA  1JG9A  1JIXA  1JM1A  1JNDA  1JNRA  1JPUA  1JU2A  1JUBA  1JZ7A  1JZTA  1K0EA  1K0MB  1K3YA  1K4IA  1K55A  1K7CA  1K7HA  1KAPP  1KBLA  1KD0A  1KEIA  1KG2A  1KHBA  1KJQB  1KPHB  1KQFA  1KQFB  1KQFC  1KQPA  1KS8A  1KUFA  1KV7A  1KWGA  1KWNA  1KYFA  1KZQA  1L6RA  1L7AA  1L8AA  1LAMA  1LFWA  1LJ8A  1LK2A  1LL2A  1LLFA  1LMLA  1LTMA  1LTZA  1LV7A  1LYVA  1LZJA  1M0KA  1M1NA  1M1NB  1M2XA  1M3KA  7ATJA | 1M65A  1M6JA  1M7YA  1ME3A  1MG7B  1MIXA  1MJUL  1MOOA  1MPXA  1MQDA  1MRPA  1MTYB  1MTYD  1MUWA  1MXRA  1N0WA  1N1BB  1N45A  1N62B  1N83A  1NC5A  1NKGA  1NLNA  1NOFA  1NQEA  1NSUB  1NTYA  1NV0A  1NVMG  1NYMA  1NYTA  1O08A  1O29A  1O4YA  1O6VA  1O98A  1OAOC  1OBBB  1ODNA  1OE4A  1OENA  1OEWA  1OFDA  1OFLA  1OFWA  1OGQA  1OGSA  1OI6B  1OI7A  1OJJA  1OJRA  1OLRA  1ON9D  1OOYB  1ORRA  1OWLA  1OX0A  1OZ2A  1P0KB  1P1JA  7FABH | 1P1MA  1PBYA  1PBYB  1PMIA  1PN0C  1PSWA  1PV5A  1PWMA  1PX5B  1PXZA  1Q0QA  1Q16A  1Q2OA  1Q33A  1Q63A  1Q6ZA  1Q7FB  1QFMA  1QGXA  1QH4A  1QH5A  1QHDA  1QHOA  1QKSA  1QMGA  1QNRA  1QOPB  1QOYA  1QSAA  1QTWA  1QUKA  1QV9A  1QW9A  1QWNA  1QWOA  1QWZA  1QXMA  1QXYA  1R0MA  1R1DA  1R3DA  1R3SA  1R4PA  1R4XA  1R5LA  1R6XA  1R89A  1RA0A  1RC9A  1RCQA  1RGYA  1RHSA  1RJDC  1RKYA  1RP0A  1RQHA  1RTQA  1RU4A  1RV9A  1RVAA  8A3HA | 1RWHA  1RX0A  1RYIA  1S0AA  1S0IA  1S3EB  1S4BP  1S95B  1S9RA  1SFSA  1SG4C  1SG6B  1SQSA  1SR4B  1ST0A  1SU8A  1SVBA  1T06A  1T0BH  1T0TV  1T1GA  1T1UA  1T2DA  1T46A  1T4BA  1T61D  1T6CA  1T6GA  1T7RA  1T9HA  1TA3A  1TBFA  1TG5A  1TG7A  1TJYA  1TKEA  1TL2A  1TN6B  1TQ4A  1TWDB  1TZPA  1U3WA  1U5UA  1U7GA  1U8FO  1U8VA  1UA4A  1UALA  1UASA  1UF5A  1UG6A  1UGPA  1UIRB  1UMGA  1UMKA  1UMZB  1UOHA  1UQ5A  1UV4A  1UWCA  8ACNA | 1UWKB  1UX6A  1UYLA  1V0EA  1V0LA  1V0WA  1V3EA  1V54A  1V54B  1V5FA  1V5VA  1V7WA  1V82A  1VAJA  1VBKA  1VBLA  1VCLA  1VIYA  1VKPA  1VLBA  1VPSB  1VYBA  1VYRA  1W0OA  1W27A  1W2FA  1W2YA  1W5FA  1W66A  1W6GA  1W7LA  1W8OA  1W96C  1WAKA  1WB4A  1WD3A  1WDDA  1WDPA  1WOFA  1WOYA  1WQ3A  1WU4A  1WUAA  1WUIL  1WUIS  1WVFA  1WYBA  1WZAA  1WZZA  1X09A  1X0CA  1X0RA  1X1NA  1X2JA  1X54A  1X6VA  1XCLA  1XDNA  1XDZA  1XFFA  9GAFC | 1XFIA  1XG4A  1XGKA  1XH8A  1XJJA  1XOVA  1XQHA  1XQOA  1XSZA  1XTTA  1XUBA  1XZZA  1Y0EA  1Y0PA  1Y3NA  1Y5IB  1Y5IC  1Y7BA  1Y8AA  1Y9GA  1YB6A  1YDIA  1YFQA  1YGEA  1YHLA  1YI9A  1YIIA  1YKDA  3JUHA  1YMTA  1YNPA  1YQZA  1YS1X  1YT3A  1Z05A  1Z0WA  1Z10A  1Z2NX  1Z32X  1Z7XW  1Z84B  1ZCJA  1ZI9A  1ZJYA  1ZKPA  1ZL0B  1ZO4B  1ZR6A  1ZUWC  1ZY7A  2A14A  2A65A  2A6ZA  2AC7A  2ACVA  2AD6A  2AE0X  2AEXA  2AFWA  2AGKA | 2AHFA  2AIJX  2AJCA  2AKAA  2AQ2B  2AQ5A  2AQJA  2ARRA  2AVDA  2AWKA  2AXQA  2AYHA  2B0TA  2B3FA  2B4HA  2B61A  3IB0A  2B82A  2BCGG  2BF6A  2BFDA  2BFDB  2BG1A  2BHUA  2BIBA  2BIIA  2BJFA  2BJKA  2BJRA  2BKXA  2BMOA  2BMWA  2BO9B  2BOGX  2BOQA  2BPTA  2BR6A  2BRAA  2BSYA  2BW4A  2BWVA  2C15A  2C1IA  2C1LA  2C1VA  2C3MA  2C4IA  2C4XA  2C5AA  2C6QB  2C71A  2C78A  2CB2A  2CB5B  2CCAA  2CDBA  2CFUA  2CGLA  2CI1A  2CITA | 2CIWA  2CK3D  2CL3A  2CN3B  2CNQA  2CTCA  2CVCA  2CXAA  2CXNA  2CYGA  2CZ1B  2D0OA  2DDSA  2DECA  2DQ6A  3BFXA  2EUTA  2F2HA  2F4MA  2F5VA  2F5XB  2F6UA  2FBAA  2FBQA  2FE8A  2FFCA  2FFUA  2FH1B  2FHFA  2FIMB  2FSQA  2FY7A  2FZVB  2G29A  2G7CB  4KNZB  2GAGA  2GAIA  2GAKA  2GDQA  2GFOA  2GK4B  2GKEA  2GSOA  2H6NB  2H7GX  2H88A  2HALA  2HFTA  2IU4B  2IUWA  2IWAA  2IXMA  2KINA  2NACA  2PGDA  2PTDA  2SQCA  3GRSA  4EUGA |
| --- | --- | --- | --- | --- | --- | --- | --- | --- | --- |

**Table S3.** A list of the PDB ids in the PDB3225 dataset.

| 4EQLA  3WQBA  4R42A  3LFPA  4BA1I  4GIOA  4OZXA  4EXOA  3QC0A  3W06A  3V46A  4AI4A  4E9OX  2Y8KA  4FVGA  3NO7A  4F3NA  3MZ2A  3RFIA  3R2VA  3WUZA  4JEJA  3UMHA  4EXRA  4PT1A  4NN2A  3RQ9A  4XEAA  4QA8A  4RLEA  4O7JA  3LIDA  3LY7A  3VN3A  3LWXA  3LHIA  4K08A  4QDJA  2YHWA  4BLRA  4B89A  3W57A  4K70A  4TYZA  4B5OA  3QZMA  4XABA  4IGKA  4NPMA  4ESQA  4CHEA  3P1VA  4D9IA  4B8EA  4H0CA  2YCDA  3LULA  3P24A  4HDLA  4U8FA  4H3WA  3TS9A  3LDCA  4B0ZA  3U9JA  3T4LA  4RTHA  3O74A  4KTYA  3NO2A  4R3HA  4HDTA  4KQPA  3VJ9A  4KOPA  4N0PA  4GB5A  4G26A  4LG8A  4HDEA  4ZEQA  4R84A  4K12B  4B4CA  4HXYA  4N5HX  4E6UA  3OHEA  4IEJA  4X9RA  4ZRXA  3ZL8A  2XRYA  4OZWA  4F0CA  4P0TA  3ZNVA  4RXLA  4H1XA  4ES8A  3TC5A  4XBAA  4HCSA  2X4KA  3ZVLA  3W9KA  5BP8A  4MYVA  4AY0A  4RFBA  4N7WA  4ACJA  2Y7BA  4HXFB  4TQ1A  3SBTA  4AK2A  5A4AA  4ACIA  4J8LA  3NDCA  4M5BA  4JVUA  3S9XA  2XSAA  4KV7A  4DEXB  4YH8B  4QT6A  5C8GA  4J27A  4YHVA  4MO7A  3LG5A  4AX2A  2XCWA  3O26A  4FWWA  4BGCA  3NRFA  4EPZA  3NDQA  3MVUA  4PNEA  4B8YA  3MC3A  4IXJA  3UEKA  4IUJA  4MI5A  4XURA  4IKVA  4EW7A  4H4NA  4LWLA  4WE2A  4LSCA  2XJPA  4ESUA  4D8BA  2YNZA  3SDBA  3M73A  3WPPA  4AR9A  4X1FA  3NVTA  4ODJA  3RQZA  4M2AA  4EL6A  2YIHA  4UC8A  4DBBA  3Q98A  3W0OA  3SHQA  4BN4A  3SC0A  4PYHA  4XKZA  2YLEA  4MPCA  4MT8A  4CD8A  3ZJEA  4PWYA  4DI9A  4G0XA  3ZBDA  3PO8A  3NMWA  3AFBA  4LBHA  4CNNA  4LXJA  3UD1A  3TGHA  3NJEA  3NQNA  4XOSA  4A4JA  3SREA  3LQNA  4YCSA  4Y04A  4APOA  4YV9A  3LI9A  3OV5A  3M6ZA  3S4KA  4PYSA  3TDZC  4KG7A  4K90B  2YA0A  3O1QA  4M7XA  4Z0VA  4MAIA  4UOBA  4RWUA  2Y0OA  4NSMA  3VSNA  4W4RA  4GNBA  4F0WA  4JDUA  4CQIA  4H27A  4I3FA  4NI2B  3LNVA  3RLGA  4MFKA  4WY4A  4R7QA  4IQHA  4KFUA  4KMDA  2Y79A  3WHJA  3OF5A  3VL1A  3QSLA  4N9WA  3M1XA  2XM5A  4NC6A  4FZVA  3V5UA  4H4VA  3QY3A  4MCRA  3PJIA  4PL9A  3MZOA  4TNNA  4H14A  3TRDA  3B0TA  4EWLA  3TJ8A  4P3VA  4KNKA  3ZVSA  4XO1A  3B0GA  2X98A  4TSHA  3OOIA  4LLOB  4YEPA  4HG2A  2XPWA  3OOXA  3UPVA  4GEKA  3N2NA  4D6QA  4CZXA  4GTVA  4G22A  4BGOA  3LEWA  4IODA  4GYJA  3MWXA  3W1OA  4RP3A  2X4JA  4MPTA  4MNOA  4R9KA  4X2RA  3S3ZA  4ZOXA  3PNNA  3RAGA  4RPMA  4R4XA  5C33A  3V7NA  3ZITA  4L3UA  3LFJA  4E1BA  2XFVA  4ROJA  3QLEA  4A4AA  4RD4A  4FQGA  3WVAA  3MDUA  2YCLA  2X5XA  4AF8A  4F03A  3V4KA  2YHGA  4R3LA  4CQHA  4R03A  4QDCA  4WK0B  4WQMA  5AK4A  4K37A  4F1JA  4KZKA  2XMIA  4INKA  4LCNA  4EO0A  3WJXA  4A35A  3MMHA  5CCLA  4R1JA  4Q7OA  2XI8A  3SCYA  4M7HA  4N7CA  4X8EA  4FZLA  3WFAA | 4HHRA  4EDKA  4DT4A  3T4DA  2X7RB  4L0JA  3M4RA  3SOYA  4U1EG  3RTAA  4CA1A  4HYQA  4M1XA  4KUKA  4YWQA  4P40A  4CRUA  3QC7A  4MMLA  4V28A  4BQHA  3UI4A  4XZDA  4CI7A  3O2JA  4RCOA  3QZBA  4JGIA  3O64A  4NUTA  3QY1A  3VU0A  4JCHA  4I3GA  3PM2A  4ATMA  3MVGA  4KV2A  4OUHA  4EHSA  3QWGA  3TC3A  4D6GA  4JNDA  4A6QA  4DO7A  4LZLA  3U99A  4JIUA  4QNDA  4M2MA  4EHCA  3W4SA  3SOJA  4E3WA  4JB7A  3NCEB  4OX5A  3U97A  4TVCA  4ZM7A  4XLGA  2XDGA  3OOSA  3WPUA  4IUWA  3TVRA  4F27A  4N0NA  4P82A  4RSLA  3TJMA  3WA1A  3R24A  3RQ4A  4GR6A  4QHJA  4QPNA  4I8IA  4IYJA  4ALZA  4N6TA  4BI8A  3M3HA  4AW7A  2XOLA  4KU0D  4N3SA  4OPCA  4H5BA  3MSWA  4LLDB  4AT7B  4L58A  3NJNA  4NAOA  4CDPA  4QRKA  4H59A  4X8QA  4GXTA  4G79A  3OSEA  4MRTA  3AL2A  3NBMA  4F0JA  3TE8A  3TBOA  3SY1A  2YHAA  3LUMA  3AKCA  3VZ9B  4CCDA  3SHGA  3LNLA  3NQIA  2XLGA  4JF3A  3OBHA  3V9OA  4AMMA  4PVKA  4FXIA  4ESWA  4ENFA  4E57A  4MLWA  4TKXL  3WWCA  4BA1A  4JD0A  3LLUA  3OIZA  3RGAA  4KCAA  4C5PA  3NYYA  3T64A  3OCRA  3SQ7A  3M66A  4BC3A  4XZAA  4V24A  3WYDA  4KX4A  4QDNA  2XDHA  4YJWA  4NV0A  3MK6A  4C97A  4DMIA  4QBNA  3MXZA  3MW8A  4GGJA  4TMDA  4Y7DA  3MD7A  3SHOA  4M20A  3M6LA  4H3UA  5AFOA  4UYPA  4XXTA  4HCHA  3ROBA  2YEQA  5C4YA  3UX2A  4Q3JA  3NCEA  4HBZA  3O70A  4KH9A  5AGVA  4HVTA  3OKGA  3WNDA  3RZVA  4XWXA  4KYUA  4PKMA  3LMZA  4LOSA  3ZX3A  2XQOA  4WKYA  4PXWA  2XUZA  3T4HB  3RT2A  4ZQAA  4YL8A  4EWEA  2YBYA  3MX7A  3UQ8A  4HLJA  3LLPA  3VMVA  4HWSA  3PE7A  4IX3A  4R2LA  4O1GA  4IAUA  4ZBGA  4AIVA  3ZUZA  3RD7A  4J7DA  4N0RA  4IYAA  3LLOA  2YANA  4YODA  4H7YA  3N4JA  5BPXA  3S0AA  4DV8A  4J2PA  3PNXA  3TQ5A  4XJ5A  3AQ2A  2YNYA  3AKAA  3OV8A  4MNCA  4YTBA  4LK9A  3NA5A  4W8QA  4A5NA  4K12A  4EFCA  4DA2A  4WY4C  4TPNA  3LQ3A  4AFHA  4U5HA  4OLTA  3QGUA  4YD8A  2XE4A  4R1DB  4LLOA  3RIXA  4GT8A  4ZW9A  4MJKA  4ERRA  4CTJA  4NN5C  4YTKA  4ZBYA  3TRGA  4IHQA  2YFRA  2X32A  4PZJA  3TG7A  4D1JA  4OA3A  3PT1A  4E5VA  4CXFA  4P09A  4JYSA  4IVKA  3MTEA  4Q7FA  4LD6A  4NUZA  3V8DA  4QB0A  3LSSA  3NVXA  3AHCA  4EYSA  4C76A  4NDAA  3VCXA  3X0TA  3M7FA  3OIOA  4JXEA  4F87A  4BPFA  4YBGA  4DKKA  4UNMA  4PMHA  4GUTB  3R4CA  4CCSA  3VORA  4RUWA  4ASMB  4EMDA  2Y4ZA  3TVZA  4EP4A  3WHNA  4O2WA  3V4GA  3SL1A  4AYLA  3PCVA  2X46A  3SGGA  4RYOA  4KUNA  4EEIA  3R1KA  3QSJA  3PU9A  3R5ZA  4L7TA  3TOQA  3WDHA  3UP3A  4MODA  4A3PA  4N5QA  3LHQA  4QP5A  3SZAA  4NMIA  4B62A  3ZRDA  3LOGA  3SX6A  3SZVA  4EUOA  3RD5A  4XEDA  4YNHA  3PF9A  4HASA  2XHAA  4IDCA  4G3FA | 4DDDA  3OIRA  4YORA  3NREA  3LB2A  3QNMA  4M5EA  3NO0A  4RMKA  4OF8A  4RG1A  3OP8A  4EW1A  4NESA  3TRCA  2XKIA  3OF7A  3SG0A  2XOCA  4MJFA  4KAVA  4ONRA  4GT6A  4ZOTA  3SHGB  4Q6BA  4YDXA  4FDBA  3WU2H  5CHSA  4AY7A  3MT0A  3PFUA  4Q1EA  4KT3A  5D2EA  3MNLA  3ZR8X  4O95A  3NA6A  4BU0A  4HH5A  4JHNA  3OCJA  4AT7A  4TVEA  3ZVWA  4O06A  5A67A  4IVQA  3W9AA  4GQMA  4R6HA  3MTWA  4FKBA  4YN1A  4W64A  4JGLA  5A9PA  3QOUA  4NHBA  4QPVA  3PP9A  3T41A  4PYAA  3SD7A  4WZXA  4I16A  3NVSA  3OTXA  4BF7A  4HFVA  3WJDA  4YF4A  5A96A  4D86A  4Q4GX  5A10A  3QW9A  4JCCA  3LURA  4UD4A  2XM2A  4N7IA  3VK0A  3QZUA  4XFMA  3OO8A  2X55A  3LWCA  4PUHA  3OVKA  4RI5A  3LFOA  3PC7A  4I4OA  3Q7MA  3LLCA  4G1IA  4C6EA  4H5IA  4BLUA  3WH1A  3UCPA  3TBDA  4P17A  4ME3A  3N1EA  4ICVA  4BWRA  3SN1A  4E4RA  3NO4A  3WKGA  4J0UA  3OQVA  3PQHA  4DEVA  4A1IA  4KQDA  4Q9BB  4BT7A  3ZPYA  3O22A  4C3XA  2XIJA  4LIXA  5BNZA  3AMRA  4ASLA  4KRGA  4K7JA  4Q98A  3OTMA  4G6XA  4PMOA  4HAHA  3MVSA  3TW0A  4D5BA  3O53A  4UITA  3O8BA  4BE3A  4KRXA  4GNEA  2Y8GA  4LW2A  3RK6A  3MALA  4MESA  3RQTA  4JP0A  4O5AA  3U7ZA  5A07A  4Y1SA  4LZKA  4L7NA  2XJ4A  3ZJ0A  5BXGA  4HS5A  3WEOA  3W7TA  4C5KA  4YIVA  4I79A  3WISA  4AFMA  4IC3A  4MSXA  3UGUA  4RHAA  4YLEA  2XHGA  4DR8A  4DIFA  3U65A  5A0LA  3R8JA  3W42A  4G32A  3Q60A  4P5NA  4BFAA  2X9LA  4GF3A  3NSWA  3VDJA  4MWIA  3TT9A  3SIBA  4WTXA  4YAHX  4E15A  3QXHA  5A2RA  3ACXA  4LHDA  4IN0A  3NV0A  4UXEA  3MDOA  5BU1A  4XFKA  3RR6A  4RA2A  5A61A  4M8KA  3NS6A  3AIAA  3S3TA  4CCVA  2X8RA  3ZIEA  3RHBA  3P3CA  3OEAA  4CJ0A  3AXBA  3TKKA  4IAMA  4QTTA  4EBGA  4N49A  4QMHA  4JIFA  4MJDA  4G5HA  3TUXA  4G9SB  4UUXA  3USHA  4YHBA  4BA0A  3QH4A  3Q3WA  3NYHA  3SQRA  4Q6TA  2Y28A  4HUTA  3TUOA  3PBTA  4KWDA  3AHNA  4CGOA  3OHGA  3MG1A  5A57A  3OH8A  3W6PA  4XVHA  4DRIB  4JTMA  3OAJA  4M1AA  4DM5A  3ZCNA  4IIYA  4CFIA  4A9VA  3T6QA  4JRFA  4MEWA  4QHEA  4YTLA  4MDWA  4IWKA  4F9DA  4I93A  3S5QA  2XBUA  3R5AA  3TTGA  3SGWA  4R0JA  5BU6A  4FTDA  2XAUA  3VAVA  4JQFA  2X9XA  4YHSA  3S25A  4UUUA  3ZJAA  3N0WA  4EHUA  3TG2A  4JBUA  5BOIA  4PV2A  3Q1XA  4KU0A  3RYBA  3NYTA  3TMGA  4G08A  3UANA  3W5FA  4OWTA  4GAKA  4Q2WA  3P02A  4UQXA  4KRUA  3PVJA  2X7RA  2YN0A  4I17A  4NN5B  4JV8B  4Q63A  3TOWA  4Y0XA  4F5ZA  4FKEA  3RHGA  3LQ9A  4M73A  3X0VA  4AYGA  4D0PA  3PC3A  3LK7A  4O8YB  4V1SA  4RW0A  4ID9A  3NRSA  4L8FA  4EICA  4GXWA  4QFLA  4MNRA  4U09A  4U6DA  4EDPA  4W4TA  4B3XA  4E6FA  4QBOA  3LCRA  3ZYPA  5BS1A  4D70A  3M6WA  2XSQA | 4DDDA  3OIRA  4YORA  3NREA  3LB2A  3QNMA  4M5EA  3NO0A  4RMKA  4OF8A  4RG1A  3OP8A  4EW1A  4NESA  3TRCA  2XKIA  3OF7A  3SG0A  2XOCA  4MJFA  4KAVA  4ONRA  4GT6A  4ZOTA  3SHGB  4Q6BA  4YDXA  4FDBA  3WU2H  5CHSA  4AY7A  3MT0A  3PFUA  4Q1EA  4KT3A  5D2EA  3MNLA  3ZR8X  4O95A  3NA6A  4BU0A  4HH5A  4JHNA  3OCJA  4AT7A  4TVEA  3ZVWA  4O06A  5A67A  4IVQA  3W9AA  4GQMA  4R6HA  3MTWA  4FKBA  4YN1A  4W64A  4JGLA  5A9PA  3QOUA  4NHBA  4QPVA  3PP9A  3T41A  4PYAA  3SD7A  4WZXA  4I16A  3NVSA  3OTXA  4BF7A  4HFVA  3WJDA  4YF4A  5A96A  4D86A  4Q4GX  5A10A  3QW9A  4JCCA  3LURA  4UD4A  2XM2A  4N7IA  3VK0A  3QZUA  4XFMA  3OO8A  2X55A  3LWCA  4PUHA  3OVKA  4RI5A  3LFOA  3PC7A  4I4OA  3Q7MA  3LLCA  4G1IA  4C6EA  4H5IA  4BLUA  3WH1A  3UCPA  3TBDA  4P17A  4ME3A  3N1EA  4ICVA  4BWRA  3SN1A  4E4RA  3NO4A  3WKGA  4J0UA  3OQVA  3PQHA  4DEVA  4A1IA  4KQDA  4Q9BB  4BT7A  3ZPYA  3O22A  4C3XA  2XIJA  4LIXA  5BNZA  3AMRA  4ASLA  4KRGA  4K7JA  4Q98A  3OTMA  4G6XA  4PMOA  4HAHA  3MVSA  3TW0A  4D5BA  3O53A  4UITA  3O8BA  4BE3A  4KRXA  4GNEA  2Y8GA  4LW2A  3RK6A  3MALA  4MESA  3RQTA  4JP0A  4O5AA  3U7ZA  5A07A  4Y1SA  4LZKA  4L7NA  2XJ4A  3ZJ0A  5BXGA  4HS5A  3WEOA  3W7TA  4C5KA  4YIVA  4I79A  3WISA  4AFMA  4IC3A  4MSXA  3UGUA  4RHAA  4YLEA  2XHGA  4DR8A  4DIFA  3U65A  5A0LA  3R8JA  3W42A  4G32A  3Q60A  4P5NA  4BFAA  2X9LA  4GF3A  3NSWA  3VDJA  4MWIA  3TT9A  3SIBA  4WTXA  4YAHX  4E15A  3QXHA  5A2RA  3ACXA  4LHDA  4IN0A  3NV0A  4UXEA  3MDOA  5BU1A  4XFKA  3RR6A  4RA2A  5A61A  4M8KA  3NS6A  3AIAA  3S3TA  4CCVA  2X8RA  3ZIEA  3RHBA  3P3CA  3OEAA  4CJ0A  3AXBA  3TKKA  4IAMA  4QTTA  4EBGA  4N49A  4QMHA  4JIFA  4MJDA  4G5HA  3TUXA  4G9SB  4UUXA  3USHA  4YHBA  4BA0A  3QH4A  3Q3WA  3NYHA  3SQRA  4Q6TA  2Y28A  4HUTA  3TUOA  3PBTA  4KWDA  3AHNA  4CGOA  3OHGA  3MG1A  5A57A  3OH8A  3W6PA  4XVHA  4DRIB  4JTMA  3OAJA  4M1AA  4DM5A  3ZCNA  4IIYA  4CFIA  4A9VA  3T6QA  4JRFA  4MEWA  4QHEA  4YTLA  4MDWA  4IWKA  4F9DA  4I93A  3S5QA  2XBUA  3R5AA  3TTGA  3SGWA  4R0JA  5BU6A  4FTDA  2XAUA  3VAVA  4JQFA  2X9XA  4YHSA  3S25A  4UUUA  3ZJAA  3N0WA  4EHUA  3TG2A  4JBUA  5BOIA  4PV2A  3Q1XA  4KU0A  3RYBA  3NYTA  3TMGA  4G08A  3UANA  3W5FA  4OWTA  4GAKA  4Q2WA  3P02A  4UQXA  4KRUA  3PVJA  2X7RA  2YN0A  4I17A  4NN5B  4JV8B  4Q63A  3TOWA  4Y0XA  4F5ZA  4FKEA  3RHGA  3LQ9A  4M73A  3X0VA  4AYGA  4D0PA  3PC3A  3LK7A  4O8YB  4V1SA  4RW0A  4ID9A  3NRSA  4L8FA  4EICA  4GXWA  4QFLA  4MNRA  4U09A  4U6DA  4EDPA  4W4TA  4B3XA  4E6FA  4QBOA  3LCRA  3ZYPA  5BS1A  4D70A  3M6WA  2XSQA | 3ZHOA  5BTYA  4EFPA  3VGNA  4K6GA  4YZZA  4X6GA  4XTVA  3QSQA  4X5MA  3AMLA  4Z24A  3V4EA  4MTMA  4R2BA  3RVCA  4HIAA  4REKA  4L9OA  3NZLA  4AZ6A  4CSRB  4RK2A  5CK4A  5AJEA  3UFEA  4E01A  4LX2A  2XZGA  4L9AA  4MDAA  4MSPA  4WY4D  3U4VA  4KTPA  4Z3XA  4QLPB  3ZNUA  4LIJA  4I6RA  4XP7A  4Q51A  3PA8A  5AX0A  3SJ5A  4ZFLA  3UWSB  4LIIA  3QI7A  4A8UA  3R0NA  4YYCA  4DJAA  4TS4A  3M7UA  4CD5A  4AE2A  4BA1B  4TVVA  3RBSA  4A37A  3RPWA  4I2NA  3QAXA  4GHNA  3VUPA  4IQMA  4XXXA  3QP3A  3OISA  3OWRA  4XB9A  4L4EA  2Y9UA  4R81A  3APQA  3VVYA  4WW7B  3PS0A  4GXBA  4KT6A  2YHSA  4EVWA  4IBNA  4GIMA  4XCVA  3OA4A  3QLIA  3NGFA  3MSTA  4LTNA  3PA6A  2YJGA  4QWTA  4XIJA  4U4PB  3NE8A  3RLEA  2X7MA  4EADA  4R75A  3TU8A  4WQKA  4Z9NA  4WXSA  3LVUA  4FN7A  3LM2A  4TJVA  4BZ7A  4WRIA  3M70A  4HBDA  4IPUA  3WJ1A  3P8TA  4M37A  3LCCA  3PJ0A  4JDYA  4XPZA  4KM6A  3MPCA  3LY0A  2XFDA  3Q64A  4F7HA  3SUMA  4FC9A  4MI7A  4HKHA  5D1IA  3THKA  4AO8A  2XZ4A  3WG7G  4L9BA  4D5RA  4R1DA  3OTNA  4R9IA  3Q1CA  3MBKA  4DPZX  3LMAA  4EOEA  4LD1A  4CN0A  4ODKA  4ZAVA  3QL9A  4FD9A  2X6WA  4GKMA  4Y0AA  4B46A  3LDUA  4IP7A  4OCVA  4ZEVA  4NMWA  3MQZA  4O7KA  4PDNA  3S6FA  3UBMA  4N6WA  4RKSA  4HVKA  3O0PA  4GUDA  3WCQA  4H7UA  4QPWA  4IUPA  3Q20A  4UYPB  4MUPA  3RHZA  3ACHA  4LRJA  5A12A  2YFDA  4R1SA  4XPLA  4N1LA  4WIQA  3MDMA  3S2JA  4P29A  4X33A  3LI5A  3ARLA  3NT1A  4NZNA  3U9GA  4CU9A  4MZDA  4MAAA  2XXPA  3RP8A  3OL3A  4X90A  4X1ZA  3AWUA  4W78A  3W5XA  3PVEA  5BP9A  3NY7A  4PP4A  4Z2OA  4KSNA  3ML1A  3MABA  4D4ZA  4JO5A  3PF7A  5AMEA  3NY7B  4EGCB  4RVQA  2Y3CA  5AGIA  3RRXA  2X3GA  3TYSA  4PS2A  3LS9A  4MO4A  3V7YA  4QC6A  4B6GA  4UT1A  3MKHA  3PJXA  4Q6UA  3MVCA  3TXSA  4I0WB  2X9JA  4NWYA  4OE9A  3LAEA  3AWMA  3ST1A  4URRA  3MILA  4OE8B  4IENA  4NDBA  3UFBA  4JJOA  3TM4A  4N2KA  3OFKA  3P2SA  4BWCB  3B02A  4PSRA  2X5FA  4ERCA  3WY8A  3ZRXA  4RP9A  4K21A  4FYUA  4EGUA  4MHXA  3ZD9A  3S6EA  3SK2A  3LUFA  3MHGA  3SCEA  4AYOA  4M23A  3PE6A  3WPQA  5CJ3A  4GC5A  3QSGA  3MHRA  4DUIA  4H9NB  4PUXA  3OA5A  3Q6DA  4MIWA  3WG3A  5BTWA  3PP5A  4REPA  4L57A  3W5SA  4E9EA  4HAMA  3MZ1A  4N8NA  3UOAB  3B1BA  4O1EA  4USOA  4NOAA  3ULJA  3NW4A  4JMPA  4Q53A  3V75A  4WBSA  3UQSA  2YHKA  4QGSA  4WTPA  3QVPA  5AWOA  3ZOGA  3S4YA  4TNDA  4QIUA  4PDYA  4TQXA  4WBTA  4BJIA  3LYHA  3WU2B  4RELA  4DMGA  3LSNA  4PWTA  2X4LA  4A0TA  4NOHA  4B1MA  4PEFA  4N6XA  4CNDA  4CKKA  3NG7X  4ICIA  3SY6A  4Z04A  3Q6SA  4G4IA  4QFTA  4P0GA  4G54A  3TR0A  4A0ZA  3ZSUA | 3UWDA  3OBEA  3TDZA  4KT3B  4S39A  4ID3A  4R6YA  4MLZA  3QZRA  4DQJA  3O9OA  4CW4A  4E1OA  4NWBA  4EBYA  4I6XA  4O8VA  3NOJA  4FR9A  4PKFA  4S28A  4N4JA  3NR5A  4P6AA  4K90A  2YMVA  4CNMA  3RKLA  3WV7A  3UEJA  4B2ZA  4W7LA  4RNXA  4EA9A  4U3EA  2X36A  4IRGA  3OWCA  3AONA  3M7OA  3P4GA  4ZK1A  4ZA9A  4ZJNA  4HFMA  4O87A  4J4HA  3NZTA  3TEWA  4GPVA  3N07A  4FNMA  4C5WA  5C0PA  4FBCA  4JG2A  4Y9TA  4EAEA  3MDPA  4OAHA  4D40A  4MV4A  4Q34A  3TBJA  2YH6A  4NLMA  4RV5A  4G3VA  3TKFA  4KWUA  4BQ2A  4EYZA  3ON2A  3NKGA  3ATSA  3O31A  3OG9A  4RCIA  4YF1A  4E1PA  3P0FA  3MCIA  3WNOA  3WWXA  4L68A  4MXPA  4LQZA  3PC6A  4ZCDA  3LP5A  4LGOA  3SGZA  3Q13A  3U3ZA  4RGUA  2XYIA  3QEKA  4IFAA  4IKNA  4MBOA  4UP4A  3NYMA  4J32B  4ACOA  4K2PA  4IGUA  4KJMA  4H5UA  4WW7A  4I0OA  4J6OA  4PFHA  4BZAA  4AE7A  4L8PA  4ORDA  3X0FA  3NNBA  5AGDA  4X4WA  3NO6A  4GOQA  4LXOA  4YUXA  4O65A  4PWQA  4I9DA  3UCJA  3AJ7A  4EFOA  4P9IA  4A2VA  4UVQA  4L2IB  4QQ4A  2XC1A  2Y08A  4CLLA  4C6SA  3WDQA  4Q7QA  2YG9A  4QWOA  4OYUA  4BWVA  4BL7B  2XHFA  4NI2A  4KE2A  4B2NA  4UOPA  4HZIA  4TX5A  3MP9A  4UZGA  4QOSA  3U6GA  4R5RA  4UNRA  3S8GA  4FS7A  4U9CA  3ZT9A  4JDNA  4PFYA  4MB4A  4FNVA  3WJPA  4WUBA  4J33A  2YKZA  4OEBA  3VQTA  4IPIA  4I51A  4IGIA  4GS3A  3MJFA  3VU9A  3AFOA  4UEXA  4EVQA  4UQWA  4PZ3A  2XIOA  4HX0A  4OMBA  4JHYA  4I4KA  4OWKA  4YNXA  4BBYA  4D05A  4IA6A  4AGKA  4Q29A  3N9BA  3NFKA  4G3QA  4JN3A  4M1HA  4DPPA  4BOUA  4OK9A  2YIZA  4JOQA  3N0UA  4GHJA  4D0QA  4QBUA  5BY8A  4KYQA  4CGQA  4O4FA  4ESPA  4HB9A  3RO1A  3TTCA  3WNVA  4BJAA  4BJ0A  4M7TA  3NKEA  4INEA  3S9ZA  3AWUB  3MZFA  4OQ1A  2XDPA  4X7YA  3VENA  4QRLA  4WKSA  3ZK4A  3ALJA  3RPDA  3LX3A  4LJ1A  3R41A  4A57A  3TP4A  4QQ0A  4AJYB  3VRDB  4LEVA  3MM4A  4GGGA  4K3ZA  4WCKA  4HFSA  4YX1A  4X33B  3NZNA  3T7DA  4OPBA  4LPQA  3ZW5A  4BB9A  4HCIA  4AUKA  4K22A  4FCJA  3QP6A  4NG0A  3OJ0A  3PMMA  3PMSA  4E1SA  3TDWA  4CHIA  3VX0A  2XWVA  4BQNA  4MUBA  4D9OA  4ODPA  4ZOYA  4ZJHA  3T8KA  4HLBA  3ONJA  3OYVA  5AECA  4TRKA  4JEDA  3B0PA  4WP6A  3L9AX  3OGAA  3M1UA  4KH8A  3MT6A  4R2YA  3AIIA  4EZIA  3M1DA  4IC9A  3UIDA  3VBCA  4TSHB  4REOA  4ZYEA  4H3VA  4IRTA  4OX6A  4K2MA  3Q0WA  4QKDA  3SIGA  3LKKA  4IZXA  3OCMA  4LS3A  4PXYA  4C4AA  3LX4A  3QYFA  3OFGA  4U1EI  3UF6A  3MW4A  4NCXA  4HTLA  3SRIA  3ZXCA  3QVXA  4LY1A  3ASLA  3LKMA  3SUKA  3RU6A  5CE7A  4AVRA  3NX4A  3OU2A  4OI3A  4DZOA  4K5SA  2X5YA  4UZ1A  4P5EA  4J42A  4NX8A  4P98A  4YSLA  3QHPA  4N6QA  3NWCA  3SMAA  2YB1A | 4OFKA  4M5RA  4YFVA  3NRHA  4HVYA  2XPPA  3ZLMA  3W08A  4IILA  3T47A  4PL6A  2X26A  3VBJA  4GBMA  3B08B  2XXNA  3LLBA  4GVCA  3SZ7A  3V4CA  4UYBA  4BI8B  4F4HA  4H2GA  3MB5A  3RNQB  4UE0A  4UU3A  4CRUB  4EGDA  4V1GA  3P2CA  3ZIBA  4LANA  3V0DA  4FYPA  3ZYQA  4L4QD  2X3MA  4ROHA  4HECA  4RZYA  4OJXA  3M1EA  2YLNA  3TO7A  3OXPA  3O12A  2XOVA  3P1GA  4I71A  4IQLA  4E3EA  3NJDA  5A0DA  3LHEA  4K1XA  4BPSA  4IHMA  3R5TA  3MVKA  3SO6A  4N8MA  4IGZA  4YYFA  3O14A  4NB5A  4P5PA  3W5NA  4C1OA  5A29A  4LYPA  4ONWA  4Y9JA  3SDOA  4OPWA  4MYZA  4O0KA  3RAUA  4NBIA  3PFEA  4BNBA  3TM8A  4CZXB  4IHEA  4HLSA  3OMTA  3AS5A  3O6NA  5CYVA  4JMDA  4MYLA  4PWOA  3LYYA  5C00A  3NTXA  4R4KA  3SMZA  4PAGA  3U8VA  3SSAA  3UENA  3V7BA  3WZ3A  3UC9A  3U3LC  4QHWA  4U36A  3MTRA  3WU2Z  4QI8A  3NEHA  3QFMA  4C0NA  4CSBA  4FFLA  4FAIA  4MXTA  4GDAA  4OMFA  4RKQA  3TJYA  3O8MA  4PMXA  3P6IA  3OBUA  5CM7A  4YTVA  4JBDA  4LHSA  4U9OA  4H4DA  4EHXA  3MJOA  4E9LA  4WWHA  3MGDA  2X5PA  5BQPA  3RFTA  4K8WA  4GPZA  3RG9A  4QJVA  3SXUA  3ZO9A  3ZIWA  4BPZA  4WH5A  4ONMA  3M07A  3VY5A  4OFFA  4S1PA  4PS6A  3MCXA  3VFZA  4XLGB  3RVAA  4EI0A  4BOLA  3VA4A  4QEGA  3AZOA  3QC5X  4Y89A  4IQ0A  3T6EL  3ML1B  4YH8A  4LGXA  4BOQA  3NH4A  3AEHA  4CYBA  4C5EA  4MN7A  4F7UG  4IGVA  4E8DA  4G1QA  4YS0A  4HO4A  4QLPA  3X34A  3SBTB  4HEOA  4KDRA  4RU1A  3TDSA  3OM0A  3Q7RA  5A98A  3NG2A  3OSRA  2XRHA  4D9SA  4MTUA  4F7UE  4WT7A  3OEPA  2YCLB  4M85A  3ND1A  4HU2A  3TLQA  4PLPA  4C2EA  2X61A  4NL9A  3LL3A  4K00A  4LZHA  4BRCA  4WQDA  4LPSA  3N01A  4GYWA  3OSQA  3O6CA  4H08A  4OFCA  4NL5A  4BL0B  3S8MA  4I4CA  3Q1NA  4PJRA  4EBJA  2XSEA  4N0HB  3Q12A  4OD8A  4JNEA  4NCRA  4JWXA  4K35A  4MZVA  4K02A  3OMDA  4Q0YA  4YTDA  3N6ZA  4G10A  4S2VA  2Y43A  4CVRA  3W6BA  4S12A  3PIWA  4OPMA  2XT2A  4JQPA  3MOZA  3MXOA  4CT3A  4JW0A  3AQJA  4N0HA  3NYCA  3ZSCA  3QF2A  2XETA  3MMGA  4NYQA  4B5SA  4J0DA  3PAJA  4H7WA  4JM1A  4DYQA  4MLVA  3PMOA  3UPSA  3SH4A  4E2UA  4U9BA  3TC8A  4Q4W1  3ADOA  4MU9A  4R1HA  4P04A  4ZO2A  4JL5A  3RF6A  4BFOA  4USXA  3UUEA  3VV1A  4ZE8A  4QCJA  3M9LA  4V23A  3PFOA  4F7UA  4NJHA  3QVQA  3UE2A  3VKWA  4I0XB  3TY1A  4PYUA  3Q9OA  4HI8A  4WASA  4XXFA  3ZOJA  4F7UP  4CXFB  4HESA  4BVQA  3OQPA  4WSFA  4FBSA  4O9EA  4INWA  3TU3B  4GQ4A  3LE4A  3TOSA  3M0ZA  3T6EH  3QBMA  4PK9A  2XTSB  3MWZA  3T6PA  3NYQA  4WN5A  3M84A  4HC5A  4KV9A  4J7AA  4J5RA  4NN5A  4E2ZA  3ZRIA  5BY1A  4X2GA  4NTCA  4XDAA  4K6NA  4PHRA  4BZ4A  4PZSA  2Y7EA  4K73A  3RPCA  5AIMA | 5CRWA  4F67A  4ZURA  4BMWA  3SLRA  4L9NA  4COKA  4E2GA  4MOVA  4ZNKA  3VZ9D  4FDTA  3MXNA  5BRLA  3VTOA  3OTIA  3MQHA  3ZRZA  4JA8A  4GMFA  4EIUA  3QU5A  2XEXA  4FAYA  3M8JA  3PMEA  4H79A  4J8SA  5A3NA  3WHRA  4WA0A  4XVVA  4GI5A  4FXQA  2Y27A  2X5NA  4ZLFA  4AFKA  4DLQA  4MPGA  4HAAA  4IYKA  4CE7A  4N30A  3T7VA  3RNLA  4HH3C  3LYDA  5C1ZA  3S2RA  3M8TA  4XFJA  4HBQA  4RGIA  3LGDA  4FB7A  4ATGA  3S81A  3ZGHA  4GWIA  4QMDA  4PWZA  2Y6UA  3WNHA  3P2UA  4RSWA  3ON9A  3S5WA  3AONB  4TR3A  3TN4A  4A0PA  3QHOA  3RKGA  3LJUX  4EO3A  4PW0A  3WMVA  4HCJA  4NI6A  4B8NA  4JR6A  3QF7A  3S8GB  4UZUA  4LMSB  3AYJA  5BRHA  3AKHA  4W8HA  3LCNA  5BMTA  4WJIA  4U98A  4F2EA  4OWTC  3WMTA  4TL6A  2YPOA  2XMRA  4DHVA  4X9CA  3U0VA  4IKDA  4WYHA  3OS4A  4L9PB  3ZN4A  3ZY7A  3LASA  4QBBA  2XIGA  4DQ6A  3RBQA  3VAAA  4I7WA  3WG7D  2YOBA  3AJRA  4M8DA  2XF3A  4M83A  4RXIA  4Q68A  4GP7A  2XZEA  4O5FA  4G29A  4UR7A  4CZUA  2XMXA  3WW3A  4LRTB  3WU2A  4LQKA  4CDJA  4E70A  4WJTA  2Y8NA  4Z3XE  5BONH  4N2XA  4CS4A  4P3HA  2YMMA  4I0WA  4O4OA  4Q3OA  4EUNA  4M6RA  2YG2A  3ZXOB  4IIBA  4HSTB  3WQCA  4MYYA  4A5UB  4G9MA  3LL7A  4PYRA  3PIJA  3TW1A  4K05A  4FVDA  3ZQKA  3T8WA  4FZOA  3N72A  4AL0A  3TR9A  3UCDA  4Y7LA  4IU2A  2X3CA  4X3KA  3ZBQA  4TPVA  4OK4A  3UMOA  4FK9A  3R9MA  5BMOA  2X1KA  4UMIA  3Q49B  4JXRA  4DELA  4BJZA  3ODGA  4MTLA  3SMVA  4R60A  3WWAA  3MUJA  4EIBA  4AP9A  4N01A  3S1TA  4EVUA  4FGQA  3ZLCA  4TZHA  4TKCA  4JYKA  3UDFA  4N3TA  4C3SA  4BHXA  3U52A  4KSYA  3T1RA  3RLSA  3PH9A  4LSBA  4MSOA  4HS1A  3ORKA  4IG1A  3N52A  4MO2A  3ON4A  4LU2A  3RV1A  5A6WC  3AFHA  4CI9A  3X0UA  2Y1BA  3LK5A  4NDSA  4R6KA  4O6MA  4GYMA  2XVYA  4NEXA  3LETA  4U5WA  4O1RA  4ZQXA  3ME7A  3UXFA  4EU9A  4I84A  4U4PA  4NUUA  4GC3A  4PE6A  3RJTA  4GEIA  3PG6A  4HI8B  3QZ0A  4C1WA  4PHJA  3QNSA  3RJUA  4ROQA  4E3YA  3OAMA  3OA8B  4G78A  4KEMA  3WHTA  4XXLA  4ZDJA  4EQAC  4JP6A  3SJLA  4I7EA  4BZPA  3LWGA  3N27A  4AGHA  4NPOA  4HT3A  3LFHA  3PFGA  4LTTA  3M3PA  4QPKA  3P9AA  3OVPA  4KQ7A  4EJQA  4UHQA  3LUUA  3MM5A  4WCXA  2XZ9A  3AUMO  4KKRA  3WVQA  3ZBGA  4RDBA  4NNOA  4UMAA  4TPSB  4TMEA  2YK4A  3O0DA  4DZIA  4ESEA  3R3QA  4OMGA  4QXBA  3PHZA  4HHVA  3NDOB  3M6JA  3O3MA  3T3WA  4HMSA  2Y4YA  4F7UF  4CITA  3O0YA  3WU2C  3TK8A  4IU2B  4GJ4A  3WXYA  3WG7C  4BVAA  4MAQA  4Q5WA  4F3VA  4WX0A  5A0NA  3SFMA  4CJ0B  3NZEA  2XTYA  2X3JA  3UTKA  3V0RA  3LG3A  3ZQOA  4ES1A  4XEPA  4R2FA  3VGIA  3VDIA  3PP2A  5A6WA  4TXDA  4G75A  4I68A  4A7WA  3P9NA  4GMQA | 4NOBA  4UQUA  3VQJA  4MAKA  4LM8A  4QFUA  4YARA  4RBRA  2XU8A  4EIVA  3LR1A  4ADNA  3SSOA  3MXNB  4CQ4A  4WFXA  3O2UA  4K7CA  4XFRA  4GUCA  3WX7A  4EKXA  4MZIA  2XOMA  4LMIA  4ML9A  4DSDA  4OJGA  4N81A  4S2RP  4DK2A  2Y53A  4KALA  4MH4A  4GVQA  4RUQA  2Y71A  3RMQA  4PD0A  4CXPA  4R9XA  4D5EA  4GCSA  4PH2A  3O1KA  3N79A  3MPRA  4OVYA  4AXOA  4Q4W2  3VC1A  3SB1A  3RHTA  4M3PA  3V5CA  4ZGMA  4BH5A  3WFIA  3UPLA  2X9OA  3M7FB  4V3IA  3ZIDA  4HF0A  3UV9A  3PJLA  4MU3A  4ZU4A  4MRTC  4N2PA  3MPBA  4A4YA  4YXPA  3O0AA  4HSTA  3TD3A  3LOPA  4ZNMA  4I6YA  3O9ZA  4N21A  5AHNA  4B2HA  4X2HB  4MGQA  3PESA  3Q2UA  4ADIA  3N0RA  3PPMA  4CRWB  4OTNA  4QJVB  3WG7H  3NBCA  3RCOA  3UWSA  4XPQA  4H9NC  4L07A  3RX9A  4YT2A  4E19A  4MKOA  3VMKA  4Q4W4  3WG7E  3VMNA  3S8IA  3WASA  4U5RA  4CSRA  4Q88A  2YC3A  3LUYA  4P3FA  5B8FA  4W9ZA  4U49A  4MUOA  4I66A  3UXJA  2XODA  4WBDA  4BEGA  4EUUA  4H6CA  3QTAA  3NFIA  3MQ0A  4EKXB  3OP6A  4YMHA  4QE0A  3TFJA  4LUNU  3LZKA  3LYGA  4OYDA  3RTLA  4NBGA  4X2HA  3NFWA  4C08A  3VK5A  4WH9A  4INDA  4D79A  4S1HA  3SNYA  4J32A  5C5ZA  4IVNA  2Y8UA  4IJ5A  4AO9A  3WURA  4I0XA  4IJNA  4O9KA  3NUFA  4PU7A  4NMYA  3LOUA  3M1TA  4DB5A  2Y1NA  4O66A  2XUAA  5AUNA  4L8AA  4U72A  3PZSA  4CFSA  3OF4A  2Y8NB  3AP1A  2XNQA  5BY8B  3N75A  4WUMA  3SP7A  4LC1A  3WEUA  4IUSA  3T92A  4RSCA  3UB1A  4FFUA  4NXIA  3MCZA  2XTSA  4CN9A  3AG7A  3O10A  2YILA  2X3HA  3M0JA  3ONDA  4BIXA  4R8XA  4NE2A  4NL9C  3U2AA  4RK6A  4KL5A  3RRIA  4IZHA  3U1WA  5AMHA  4D3DA  4XB6D  4AZJA  4JJ0A  2X2SA  4OKZA  4BL7A  4BGPA  4UWXA  3OA8A  4F7UB  4BWCA  4AQ6A  4H41A  4AXIA  4DF1A  3LIYA  3P0BA  4RG2A  4COGA  4O1WA  4HRGA  3U1DA  4P3AA  4XD1A  4ESKA  3MSUA  3QXZA  4J4RA  4NY7A  3MR0A  4R8HA  2XUVA  4W1TA  4P3XA  3U7RA  2YFUA  3L8QA  3S1XA  3SBQA  3O94A  3U52C  4UYIA  3LM4A  4Q4W3  2XW7A  4ILFA  4LVFA  4CE8A  4J3GA  3TGNA  4WESA  4OWTB  4MI4A  4FS8A  4XB6C  3U1LA  3LD7A  3ZX4A  4XB6A  4RHSA  4PQAA  4ATHA  3PDUA  3OJ6A  4Y0HA  3MI2A  4F8KA  4KT6B  3WG7F  4L2IA  3S06A  4P0ZA  3MGKA  4AK5A  4Y0GA  2XSUA  4XB6B  4FQNA  3SLZA  3OUGA  4LQBA  4HH3A  4HKEA  5A3AA  4LQ6A  4B2OA  3ZG1A  5AJJA  4PJ2A  3QITA  3PPLA  4YWAA  3URRA  3ANOA  4HFQA  4XDZA  4Q1KA  4H0SA  3T9GA  3N0KA  4XQAA  4X84A  3TBNA  4OJ5A  4X00A  4RITA  4MUVA  3ON1A  4XDIA  4E38A  3PWFA  3LRTA  4PIBA  4QQ6A  3SXUB  4B4UA  4A91A  3V1VA  3NRVA  3ZRQA  4H89A  4AJYC  2YCEA  2XCJA  3O3MB  3MM5B  5A35A  3U52E  4HE6A  3VPLA  3SOEA  4DKCA  2Y32A  4QXBB  3RWNA  4NBGD  2X5CA  3TGVA  4DZ4A | 4TX1A  3TNVA  4N0HF  3WU2O  4W78B  5CR5A  4KTBA  4IAJA  3PL2A  4GT2A  4E0AA  3VCAA  3NOQA  3AYVA  4UDGF  4EVYA  4L63A  4F23A  3RPPA  4BLPA  4AVSA  3QDLA  3WQBB  3VPBA  2XW6A  4F2LA  4OMFB  4RT5A  3OP9A  3OKXA  4B8XA  2YIMA  2XBLA  4PXEA  4CNKA  3UCSA  3VRDA  4PKFB  4UM7A  3TU3A  4QUSA  3T6EC  3WU2V  4UF7C  4OD9A  4CRHA  4GM6A  4XLZA  4BT9A  4DOYA  3O48A  3UUWA  4OMFG  3Q3MC  4DPLA  3WSGA  3SJLD  3WHRB  3PF6A  3PLWA  3N6YA  3N29A  2XWSA  4R2XA  3WU2U  3Q3MB  3M0MA  3AJ4A  4AUMA  4HWXA  3WG7I  4LMSA  3OGHA  3N9UC  4C9SA |
| --- | --- | --- | --- | --- | --- | --- | --- | --- | --- |

**Table S4.** A list of the PDB ids in the MoDEL136 dataset.

| 1EW4A  1BUYA  1XSMA  1J7XA  1G43A  1XR6A  1AF4A  1NREA  1AIRA  1BM8A  1JHSA  1A27A  1CEIA  1GDFA | 1GPCA  1C3DA  1JUDA  1D2PA  1H3QA  1O5TA  3SSIA  1A62A  1GENA  1AQEA  1A1XA  1SFPA  1FBRA  1AUAA | 1BFEA  1SKZA  1UTGA  1A87A  1E5BA  1EW0A  1IFGA  1TZQA  1DABA  1ANUA  1A32A  1OTPA  1C25A  1QLMA | 1AHQA  1DOVA  1KNBA  1A43A  1CBYA  1HQ0A  1A0KA  16PKA  1FIBA  1C05A  1J57A  1UOKA  1IB8A  1AKOA | 1NG6A  1CIYA  1HUSA  1K04A  1H6LA  1KP6A  1PJBA  1HCZA  1OBRA  1WHPA  2ENDA  1A44A  1QAZA  1AEPA | 1MSCA  1CHVS  1A2PA  1AD6A  1EIFA  1VE0A  1TIGA  1A17A  1JLNA  1WHZA  1F2HA  1T71A  1N81A  1O6WA | 1LBUA  1LXAA  1PKPA  1PILA  1AK2A  1A23A  1AKDA  1MLAA  1LMIA  1RFSA  1FI2A  1A1WA  1H8MA  1A7WA | 1S2OA  1A8QA  1A7GE  1RISA  1B11A  1L7LA  1UORA  1J7GA  1LPLA  5HIRA  1B5MA  1CDZA  1KVVA  1KKHA | 1DT4A  1AS0A  1XWLA  1VIEA  1DV8A  1EH1A  1FWPA  1DXJA  1D1LA  1AMXA  1RMDA  1FTSA  1EYHA  1ABVA | 1ADDA  1FX4A  1EIAA  1MSKA  1NE9A  3EZMA  2PIAA  1BOLA  1PGSA  1AKQA |
| --- | --- | --- | --- | --- | --- | --- | --- | --- | --- |
